# Sequence- and structure-specific cytosine-5 mRNA methylation by NSUN6

**DOI:** 10.1093/nar/gkaa1193

**Published:** 2020-12-16

**Authors:** Tommaso Selmi, Shobbir Hussain, Sabine Dietmann, Matthias Heiß, Kayla Borland, Sophia Flad, Jean-Michel Carter, Rebecca Dennison, Ya-Lin Huang, Stefanie Kellner, Susanne Bornelöv, Michaela Frye

**Affiliations:** Department of Genetics, University of Cambridge, Downing Street, Cambridge CB2 3EH, UK; Department of Biology and Biochemistry, University of Bath, Claverton Down, Bath BA2 7AY, UK; Washington University School of Medicine in St. Louis, 660 S. Euclid Ave, St. Louis, MO 63110, USA; Department of Chemistry, Ludwig-Maximilians-University Munich, Butenandtstr. 5-13, Haus F, 81377 Munich, Germany; Department of Chemistry, Ludwig-Maximilians-University Munich, Butenandtstr. 5-13, Haus F, 81377 Munich, Germany; German Cancer Research Center – Deutsches Krebsforschungszentrum (DKFZ), Im Neuenheimer Feld 280, 69120 Heidelberg, Germany; Department of Biology and Biochemistry, University of Bath, Claverton Down, Bath BA2 7AY, UK; Cambridge Institute of Public Health, University of Cambridge, Forvie Site, Robinson Way, Cambridge CB2 0SR, UK; Department of Genetics, University of Cambridge, Downing Street, Cambridge CB2 3EH, UK; Department of Chemistry, Ludwig-Maximilians-University Munich, Butenandtstr. 5-13, Haus F, 81377 Munich, Germany; Wellcome – MRC Cambridge Stem Cell Institute, University of Cambridge, Puddicombe Way, Cambridge CB2 0AW, UK; German Cancer Research Center – Deutsches Krebsforschungszentrum (DKFZ), Im Neuenheimer Feld 280, 69120 Heidelberg, Germany

## Abstract

The highly abundant N6-methyladenosine (m^6^A) RNA modification affects most aspects of mRNA function, yet the precise function of the rarer 5-methylcytidine (m^5^C) remains largely unknown. Here, we map m^5^C in the human transcriptome using methylation-dependent individual-nucleotide resolution cross-linking and immunoprecipitation (miCLIP) combined with RNA bisulfite sequencing. We identify NSUN6 as a methyltransferase with strong substrate specificity towards mRNA. NSUN6 primarily targeted three prime untranslated regions (3′UTR) at the consensus sequence motif CTCCA, located in loops of hairpin structures. Knockout and rescue experiments revealed enhanced mRNA and translation levels when NSUN6-targeted mRNAs were methylated. Ribosome profiling further demonstrated that NSUN6-specific methylation correlated with translation termination. While NSUN6 was dispensable for mouse embryonic development, it was down-regulated in human tumours and high expression of NSUN6 indicated better patient outcome of certain cancer types. In summary, our study identifies NSUN6 as a methyltransferase targeting mRNA, potentially as part of a quality control mechanism involved in translation termination fidelity.

## INTRODUCTION

Over 170 known RNA modifications extensively increase the functional diversity of RNA molecules (1). Accordingly, RNA modifications emerged as an important additional regulatory layer of gene expression programs and are often required for normal development (2). Recent advances in detection technology, mostly associated with high-throughput sequencing, have revealed how RNA modifications influence many stages of RNA metabolism, and thereby effect diverse biological processes including cell fate decisions, immune responses, and tumorigenesis (3–5).

One of the best-studied modifications is N6-methyladenosine (m^6^A), the most abundant internal mRNA modification that controls gene expression (6). The prevalence and function of other rarer mRNA modifications such as N1-methyladenosine (m^1^A), N6,2′-*O*-dimethyladenosine (m^6^Am), m^5^C, 5-hydroxymethylcytosine (hm^5^C), and pseudouridine (Ψ) are less well-characterised and often somewhat controversial (7). One of the most disputed modifications in mRNA is m^5^C as current conventional detection methods are associated with high background levels, making this low-abundance modification particularly challenging to define (8,9). Furthermore, the total levels of m^5^C in mRNA varies between tissues (10). Nevertheless, NSUN2, a m^5^C RNA methyltransferase mainly targeting tRNAs, has consistently been linked to mRNA methylation (11–15).

NSUN2 is one of eight evolutionary conserved m^5^C RNA methylases (NSUN1–7 and DNMT2) (2). Of these, NSUN2, 3, 6 and DNMT2 have all been shown to methylate tRNAs, yet in a non-overlapping and site-specific manner (15–20). NSUN2 is however the only enzyme with a broader substrate-specificity, methylating the majority of expressed tRNA, other abundant non-coding RNAs and a small number of mRNAs (11–15,21).

Here, we map NSUN6-dependent m^5^C sites in RNAs in the human transcriptome using our recently developed miCLIP method (12,22). Unexpectedly, we find that most sites located to protein coding RNAs within the consensus sequence motif CTCCA. NSUN6-specific sites were enriched in the 3′UTR and marked translation termination. RNA bisulfite (BS) sequencing confirmed m^5^C sites in mRNAs that were lost in knockout cells and rescued by over-expressing the NSUN6 protein. NSUN6-targeted mRNAs were more abundant with higher translation rates. NSUN6 was dispensable for mouse embryonic development. However, NSUN6-expression levels were down-regulated in tumours when compared to normal tissues, and high NSUN6 positively correlated with patient survival rate. Together, our study shows for the first time that NSUN6 mediates site-specific deposition of m^5^C in mRNA, potentially as part of a novel translation quality control mechanism.

## MATERIALS AND METHODS

### Cell culture

The human embryonic stem cell (hESC) line Hues9 (H9) was obtained from the Wicell Research Institute (Madison, WI) and maintained in Essential 8 media (Thermo Fisher Scientific) on hESC-Qualified Matrigel (Corning) coated plates. Media was refreshed daily and the cultures were dissociated in clumps every 4 days using 0.5 mM EDTA in PBS. In the embryoid bodies experiments, 70% confluent hESC were dissociated in clumps and seeded on ultra-low attachment well plates (Corning) maintaining a 1:1 dilution factor. After plating, hESC were cultured in Essential 6 media (Thermo Fisher Scientific) plus 10 μM rock-inhibitor (Y-27632) (Stem Cell Technologies Canada) for the first 24 h and for further 7 days in Essential 6 media (Thermo Fisher Scientific).

HEK293 (HEK) (ATCC) and MBA-MD-231 (ATCC) cells were grown in DMEM Media (Thermo Fisher Scientific) supplemented with 1mM Glutamax (Thermo Fisher Scientific), 10% heat inactivated FBS (Thermo Fisher Scientific). All cells were grown at 37°C, 5% CO_2_.

### Migration assays

NSUN6 was knocked down in MDA-MB-231 using NSUN6 siRNA pools (siTOOLsBIOTEC) via RNAimax transfection reagent (Thermo Fisher Scientific), according to manufacturer's instructions. Twenty-four hours post transfection, NSUN6 and control siRNA treated cells were cultured for another 24 h in DMEM containing 1% heat inactivated FBS. On the day of the experiment, 5000 starved cells were plated onto a Transwell insert (Costar, 8μm pores) in DMEM containing 1% heat inactivated FBS. The plate well was filled with DMEM containing 10% FBS to promote migration. After 24 h, Transwell inserts were fixed and stained with a solution of Crystal Violet in 80% Methanol for 10 min, followed by three washes in PBS. Non-migratory cells, on the upper side of the membrane, were removed by gently swiping with cotton swabs. Cells that migrated to the lower side of the Transwell were quantified by microscopy and counted using ImageJ software. At least three representative images were taken per Transwell filter. Each sample was tested in quadruplicates.

### Generation of NSUN6 knockout, rescue and overexpressing lines

NSUN6 knockout H9 cells were generated either by using homology directed recombination (HDR) or by inserting random Indels in response of double strand breaks (NHEJ). For HDR, we targeted exon 2 of NSUN6 with wild type Cas9 (pSpCAs9(BB)2A-GFP) plus the recombination vector pD07 (Genecopoeia) carrying the selection genes puromycin and eGFP under control of EIF1a promoter and with homology arms on Introns 1 and 2. gRNAs were designed using the gRNA design software from the Feng Zhang lab at MIT (Exon2 gRNA1: ATT TTT CAC ATG TTG TAC TG **AGG**, Exon2 gRNA2: GAT GAA CTT CAG AAG GTT TG **TGG)**. Four days after nucleofection with the AMAXA Nucleofector Kit (Lonza), we applied puromycin selection until we observed the appearance of green colonies, which were screened for integration of the recombination cassette and for the presence of NSUN6 exon 2. For NHEJ, we targeted the exon 9 of NSUN6 with wild type Cas9 (pSpCAs9(BB)2A-GFP) (Exon 9 gRNA: ATC CAG AAG AAT TCG GTC AA **AGG**), 3 days after nucleofection, the targeted cells were sorted and re-plated at low density in E8 conditioned media. We then screened by Sanger Sequencing the grown colonies for InDels in NSUN6 exon 9. For generating NSUN6 overexpressing H9, NSUN6 CDS was PCR amplified from the TrueORF pCMV-Entry vector (Origene) and cloned via Gibson assembly (NEB) into the destination piggybac vector pBPCAG-cHA-IN (kindly provided by Austin Smith). This vector and the piggy bac transposase were then nucleofected into H9 using the AMAXA nucleofector kit (Lonza). Puromycin selection started 4 days following nucleofection and the surviving clones were screened by qPCR and Western Blot for NSUN6 expression. The NSUN6 knockout HEK293 cells were generated employing a similar NHEJ-based methodology, using the guide RNA sequence: CTG ATG ACA TAC TTC AGT TC. The same overexpression strategy used in H9 was also adopted to rescue NSUN6 expression in knockout HEK293 cells.

### Luciferase reporter assay

NSUN6 knockout and overexpressing HEK cells were seeded in a 24-well plate and after 24 h transfected with either the ANGEL1–3′UTR-luciferase vector (GeneCopoeia, HmiT006021-MT05) or the control-luciferase vector (GeneCopoeia, CmiT000001-MT05) using Lipofectamine2000 according to the manufacturer's instruction (Thermo Fisher Scientific). After 48 h, the medium was collected and the luciferase assay was performed with the Secrete-Pair Dual Luminescence Assay Kit according to the protocol (Genecopoeia). To quantify the reporter activity, we first normalized for transfection efficiency using secreted Alkaline Phosphatase (SEAP), which is encoded on the same plasmid. Second, we measured the relative luciferase activity from the reporter containing the *Angel1* 3′ UTR sequence compared to the reporter without the 3′ UTR (CmiT000001-MT05).

### Measuring global protein synthesis with OP-puromycin

FaDu cells were reverse transfected with a siPOOL designed against NSUN6 or a scrambled control siPOOL (siTOOLs Biotech). After 48 or 72 h, cells were treated with OP-puromycin for an hour. Afterwards, the cells were collected, fixed and permeabilized. Using Click-it chemistry (Thermo Fisher Scientific), the OP-puromycin containing peptides were stained with Alexa Fluor 647 and analysed on a FACSCanto Cell Analyzer (BD Biosciences). Cycloheximide treated cells were used as a control.

### Mass spectrometry

NSUN2, NSUN6 and DNMT2 were depleted by transfecting the corresponding siRNA Pools or a Negative Control siRNA pool (siTOOLsBIOTECH). The transfections were carried out using Lipofectamine RNAiMAX transfection reagent (Thermo Fisher Scientific) in six-well plates, according to manufacturer's instructions. The siRNAs were designed to target the transcripts coding for NSUN2 (NCBI Gene ID: 54888), NSUN6 (NCBI Gene ID: 221078) and DNMT2 (NCBI Gene ID: 1787). For sample collection, cells were directly lysed in Trizol (ThermoFisher Scientific) and total RNA was extracted according to the manufacturer's instruction. mRNA was purified in HEK wild-type as well as NSUN6 knockout and rescued cells using DYNAL Dynabeads mRNA purification kit (ThermoFischer Scientific) using half the beads and reagents recommended by the manufacturer. The resulting poly A enriched RNA was further purified with RiboMinus Eukaryote kit v2 (ThermoFischer Scientific) according to manufacturer's instruction followed by ammonium acetate precipitation. Mass spectrometry to quantify m^5^C was performed as described previously (10,23).

### Generation of the Nsun6 knockout mice

All mice were housed in the Wellcome Trust-Medical Research Council Cambridge Stem Cell Institute Animal Unit. All mouse husbandry and experiments were carried out according to the local ethics committee under the terms of a UK Home Office license P36B3A804 and PPL70/7822.

Two embryonic stem cell lines containing a knockout first allele (with conditional potential) were obtained from EuMMCR (*Nsun6*^tm1a(EUCOMM)Hmgu^). Mice homozygote for the targeted trap allele was used to analyse *Nsun6* total knockout. Genotyping primers were: *Nsun6*-forward (AAT CCA GCA TTC CTG TTG TTC AGC), LoxR (TGA ACT GAT GGC GAG CTC AGA CC), *Nsun6*–5′arm-2 (ACA GTG AGT CAG GTG AGG TGT GCC), and *Nsun6*-rev (CAC AAT GAG ACA GCA CCC AG). The LacZ-neo cassette was used as reporter for *Nsun6* RNA expression in wild-type and *Nsun6* total knockout mice. LacZ staining on whole mounts and sections of embryos was performed as described previously (24).

### RT-qPCR and western blotting

Total RNA was extracted using TRIZOL (Thermo Fisher Scientific) according to manufacturer instructions. Reverse transcription was performed using SuperScript III Reverse Transcriptase (Thermo Fisher Scientific) and random primers (Promega). Quantitative PCR were run using TaqMan probes (Thermo Fisher Scientific) for eukaryotic 18S rRNA (X03205.1), MARCKSL1 (Hs00702769_s1), TRAF7 (Hs00260228_m1), ANGEL1 (Hs00380490_m1), CALM3 (Hs00968732_g1), BAG6 (Hs00190383_m1), CUX1 (Hs00738851_m1), TRIMM50 (Hs01390531_m1), BUB3 (Hs00945687_m1), EEF (Hs00265885_g1), MACF1 (Hs00201468_m1), DLX5 (Hs01573641_mH), DNMT3B (Hs00171876_m1), FOXD3 (Hs00255287_s1), GATA6 (Hs00232018_m1), HOXA1 (Hs00939046_m1), NANOG (Hs02387400_g1), POU5F1 (Hs03005111_g1), TDGF1 (Hs02339497_g1).

For protein isolation, cells were first rinsed with PBS and lysed in ice-cold RIPA buffer (50 mM Tris–HCl pH 7.4, 1% NP-40, 150 mM NaCl, 0.1% SDS, 0.5% sodium deoxycholate). RIPA was supplemented with cOmplete Mini EDTA-free Protease Inhibitor Cocktail tablets (11836170001, Roche). Cells were collected using a cell scraper and the lysates were centrifuged for 15 min at maximum speed in a pre-cooled centrifuge at 4°C, and their supernatant collected and kept on ice. Cell protein lysates were mixed with NuPAGE LDS Sample Buffer (4X) (NP0007; Invitrogen) and run on polyacrylamide gels. Proteins were transferred to a nitrocellulose or PVDF membrane (GE Healthcare). Membranes were blocked for a minimum of 1 h at room temperature in 5% (w/v) non-fat milk or 5% (w/v) BSA (A4503–50G; Sigma Aldrich) in TBS-T (1× TBS and 0.1% Tween-20) and then incubated with primary antibody in blocking solution overnight at 4°C. Each membrane was washed three times for 10 min in TBS-T prior to incubation with the appropriate Horseradish peroxidase (HRP)-labeled secondary antibody (1:10 000) in TBS-T at room temperature for 1 h. After washing, the antibodies were detected by using the Amersham ECL Prime Western Blotting Detection Reagent (RPN2232; GE Healthcare). The primary antibody was NSUN6 (1:500, 17240-1-AP, Proteintech). Anti-γ-Tubulin (T6557; Sigma Aldrich), Anti-HSP90 (sc-13119; Santa-Cruz) or Ponceau staining served as loading controls.

### miCLIP

Full-length cDNA constructs for NSUN6 in the pCMV6-Entry-Myc vector were obtained from OriGene. Site-directed mutagenesis to generate the miCLIP-mutants was performed using the QuikChange II Site-Directed Mutagenesis Kit from Agilent as per the manufacturer's instructions. To generate the NSUN6 miCLIP mutant, cysteine 326 was mutated to alanine using the following primers: (forward) GAA TTC TTC TGG ATG CAC CCG CTA GTG GAA TGG GAC AGA GAC; (reverse) GTC TCT GTC CCA TTC CAC TAG CGG GTG CAT CCA GAA GAA TTC. HEK293 cells were transfected with either NSUN6 wild-type and the miCLIP-mutant construct using Lipofectamine 2000 (Life Technologies) and harvested 24 h later.

The harvested cells were lysed in lysis buffer consisting of 50 mM Tris–HCl pH 7.4, 100 mM NaCl, 1% NP-40, 0.1% SDS, 0.5% sodium deoxycholate. Lysates were then treated with high concentration of DNase and low concentration of RNaseI to partially fragment RNAs. Lysates were cleared by centrifugation at 13 000 rpm for 15 min at 40°C and then incubated with Protein G Dynabeads (Life Technologies) in the presence of an anti-Myc antibody (9E10, Sigma). Following stringent washing, 3′ end dephosphorylation was performed with T4 polynucleotide kinase (New England Biolabs) before addition of a pre-adenylated linker using RNA ligase (New England Biolabs). 5′ end labelling was then performed using T4 PNK and ^32^P-ATP before protein–RNA complexes were eluted and run on denaturing gels. Nitrocellulose transfer was performed, and the radioactive signal was used to dissect nitrocellulose pieces that contained NSUN6-partially digested RNA complexes. RNA was recovered by incubating the nitrocellulose pieces in a buffer containing Proteinase K and 3.5 M urea. Next, reverse transcription was performed using oligonucleotides containing two inversely oriented adaptor regions separated by a BamHI restriction site. cDNAs were size-purified on TBE–urea gels before being circularized by CircLigase II (Epicentre). Circularised cDNAs were then annealed to an oligonucleotide complementary to the BamHI site and then BamHI digested. Linearized cDNAs were then PCR-amplified using primers complementary to the adaptor regions using 25 cycles of PCR. Libraries were then subjected to high-throughput sequencing using the Illumina HiSeq 2000 platform.

### RNA bisulfite conversion and sequencing

Total RNA from H9 wild-type (WT), knockout (KO), overexpression (OEX) and HEK293 WT, KO and NSUN6 rescue (RES) cells was first extracted using Trizol (Thermo Fisher Scientific) and subsequently treated with DNaseI (Ambion) and RiboZero (Illumina) to remove contaminating DNA and ribosomal RNAs. The remaining RNA was then converted as previously described (15,25). Briefly, 10 μg of RNA was resuspended in 10 μl of RNAse free water and mixed with sodium bisulfite pH 5.0 (42.5 μl) and DNA protection buffer (17.5 μl) (EpiTect Bisulfite Kit, Qiagen). The deamination reaction was then carried out by incubating in a thermal cycler for four cycles of 5 min at 70°C followed by 1 h at 60°C and then desalted with Micro Bio-spin 6 chromatography columns (Bio-Rad). RNA was desulphonated by adding an equal volume of 1 M Tris (pH 9.0) to the reaction mixture for 1 h at 37°C, followed by ethanol precipitation. The bisulfite-converted RNA quality and concentration were assessed on a Bioanalyzer 2100 RNA nano-chip (Agilent). About 120 ng of bisulfite-converted RNA were used to generate Bisulfite-seq libraries using the TruSeq Small RNA preparation kit (Illumina). Before library preparation, the fragmented RNA wasend-repaired with T4 PNK and Spermidine (New England Biolabs). The size selection step was omitted, as the bisulfite-converted RNA was sufficiently fragmented by the previous conversion reaction. First the Illumina RNA adapters were then ligated, reverse-transcribed at 50°C for 1 h with SuperScript III and 1 mM of each dNTP (SuperScript III cDNA synthesis kit, Invitrogen) followed by 18-cycle PCR amplification.

### RNA sequencing and ribosome profiling

Total RNA extraction, ribosome profiling (Ribo-seq) and libraries for H9 NSUN6 WT and KO cells (at least four replicates each) were performed as described before (26–28). The samples were multiplexed and sequenced on the HiSeq 4000 platform (Illumina).

### Processing, mapping and quantification of RNA-seq and Ribo-seq reads

The H9 RNA-seq data was sequenced as paired-end 2 × 150 nt and the HEK RNA-seq data was sequenced as single-end 50 nt. All Ribo-seq data was single-end 50 nt. To process the data, Trim galore! (https://github.com/FelixKrueger/TrimGalore) with parameters ‘--stringency 6 -e 0.1’ and ‘--paired’ for the paired-end data was first used to remove Illumina adapters and to exclude trimmed reads shorter than 20 nt. Alignment was done using Tophat2 (v2.1.0) using an index with known transcripts (Gencode v23) as guidance and with novel splice junctions permitted. The RNA-seq reads were aligned directly to the reference genome (hg38). Ribo-seq reads were first aligned to a set of known rRNA and tRNAs (downloaded from the UCSC RepeatMasker tracks), followed by alignment of all unmapped reads to the reference genome. Multi-mapping read were excluded. FeatureCounts was used to quantify the number of reads per gene using the Gencode v23 gene models. Only reads aligning to the sense strand of the gene, represented either by its exons (RNA-seq) or its coding sequence (Ribo-seq), and with mapping quality at least 20 were counted. For the paired-end RNA-seq the additional flags ‘-p -B -C’ was specified to exclude chimeric reads and/or reads mapping with only one end.

### RNA-seq and Ribo-seq differential expression analyses

Differential expression analysis was done using the R Bioconductor edgeR package. Genes with mean expression below 1 count per million mapped reads were considered non-expressed and excluded from the analysis. The R Bioconductor cqn packages (29) was used for conditional quantile normalization to calculate offsets correcting for gene lengths (from featureCounts) and GC content (from biomaRt). The offsets were passed to the edgeR DGEList object, followed by a likelihood ratio test using glmFit and glmLRT. Five knockout cell lines (four H9 and two HEK) were compared to their respective control cell line (two H9 and one HEK). Each cell line was sequenced in four replicates.

Differential translation efficiency (TE) analysis was done with DESeq2 due to its ability to report the standard error (SE) associated with each log_2_ fold change (log_2_ FC). First, Nsun6 knockout was compared with control using the RNA-seq or Ribo-seq data separately to estimate log2FCs and SEs for each gene and assay. Translational efficiency of a gene can be defined as the difference between log_2_ FC_Ribo_ and log_2_ FC_RNA_ and the associated *P*-value is the probability that this difference is zero. Assuming that the log_2_ FCs follow a normal distribution, the *T*-statistic was calculated as $T\ = \frac{{| {log2F{C_{Ribo}} - log2F{C_{RNA}}} |}}{{\surd ( {SE_{Ribo}^2 + SE_{RNA}^2} ),{\rm{\ }}}}$. The resulting two-tailed *P*-values were further corrected for multiple testing by false discovery rate correction using the *R* function p.adjust.

### Determining read periodicity and codon enrichments using Ribo-seq

Codon usage in the Ribo-seq data was calculated following (28). The analysis was focused on reads of length 27–29, which were showing the strongest periodicity. Position 12–14 were determined to correspond to the codon at the P-site.

The bam file for each sample with uniquely aligned reads was converted to bed format. Bedtools intersect was used to select reads with at least 50% overlap to Gencode-annotated coding sequences. Next, the reading frame of the 5′ end of each read was determined using the frame information in the Gencode annotation. If the frame did not agree with the expected reading frame for that read length, the read was discarded. Then, nucleotide positions 1–27 were extracted from each read as nine codons, numbered as codon position –5 to +3, where 0 corresponded to the A-site. As expected, position –4 to –2 and +1 to +3 correlated well to the genome-wide distribution of codons in the human translatome, whereas counts from the predicted P-site and A-site did not.

The number of codon occurrences were counted separately for each ribosome-protected codon position and converted into a fraction of the total number of codons. Normalized codon counts were obtained by dividing the codon fraction at a specific position by the mean fraction across all nine positions.

### Processing and mapping of miCLIP reads

In order to reduce amplification bias, the primers used for reverse transcription during miCLIP experiments were designed to include a 6-nucleotide random barcode at positions 1–3 and 8–10 to enable tracing of individual cDNAs. Reads were de-multiplexed using the experimental barcode at positions 4–7, and reads with identical random barcodes, representing PCR products, were filtered. The number of different random barcodes for each unique read, which represented cDNA counts, was stored for further analysis. Barcodes were trimmed from the 5′end, and the adapter sequence ‘AGATCGGAAGAGCGGTTCAG’ from the 3′end of the reads with *cutadapt* (https://code.google.com/p/cutadapt; options: ‘-O 4 –e 0.06’), and only reads with a minimal length of 18 nt were retained.

Trimmed miCLIP reads were mapped to the human reference genome (UCSC GRCh37/hg19) by using *bowtie* (http://bowtie-bio.sourceforge.net/index.shtml) with parameters *‘-m1 -v1--best --strata*’ to select uniquely mapping reads allowing one mismatch. Methylation sites were thus inferred from miCLIP read truncation positions by assigning the read counts to the closest cytosine within ±2nt of the truncation site. Pooled read counts per cytosine were normalized per million uniquely mapping reads (RPM). If not stated otherwise, only high-confidence methylation sites with normalized read counts >50 RPM in at least two out of three replicates were selected for down-stream analyses.

### Quantifying coding sequence annotation and Ribo/RNA-seq reads at miCLIP sites

To visualize coding sequence across miCLIP sites, annotated coding sequences (Gencode V28) were merged using bedtools merge and was then converted to bigWig format using the UCSC bedGraphToBigWig tool. This was done separately for both strands. Furthermore, to allow visualization of RNA-seq and Ribo-seq read coverage at miCLIP sites, deepTools bamCoverage was used to convert the aligned reads to bigWig format. Each strand was quantified separately, and a blacklist file containing all rRNA, tRNA, snoRNA, snRNA and miRNA regions was provided. The bin size was set to 1 and an offset of 12 was used to only consider a single nucleotide corresponding to the ‘P’ site predicted from each read.

Next, deepTools computeMatrix in ‘reference-point’ mode with parameters -b 1500 -a 1500 --missingDataAsZero’ was used to extract annotation or reads coverage across miCLIP sites. The Ribo-seq read coverage was used to classify the miCLIP sites as start, middle and end of translation, based on the difference between the number of covered bases up-stream and down-stream of the miCLIP site. The standard error (SE) of the differences was calculated and a threshold of **±**1.96SE was used to define sites with ‘lower’ or ‘higher’ upstream signal. The remaining sites were classified as ‘unchanged’. Custom R scripts were used to combine sites on both strands and to visualize it as a heatmap or a profile plot. The scripts related to this analysis are available at https://github.com/susbo/Selmi-et-al-scripts.

### Calculating overlap between miCLIP sites and differentially expressed genes

Out of the 15,885 genes expressed in HEK, 1,853 genes were defined as down-regulated and 2008 as up-regulated at *P*_adj_ < 0.05 and abs(log_2_FC) > 0.5 in both HEK knockout clones. Next annovar (30) was used to identify the closest genes for all 252,135 putative miCLIP sites. The set of genes with exonic miCLIP sites (at 0, 0.5, 1, 3, 5, 10 or 50 RPM) was compared with genes without exonic miCLIP sites. The relative enrichment or depletion of gene down- or up-regulation was calculated as an odds ratio with a 95% confidence interval using Fisher's exact test.

### Processing and mapping of BS-seq reads

The BS-seq was processed as described previously (21). First, Trim Galore! (v0.4.0) with parameters ‘--stringency 3 -e 0.2 -a TGGAATTCTCGGGTGCCAAGGA’ was used to remove sequencing adapters and exclude reads shorter than 20 nucleotides. Next, alignment to the hg38 reference genome was done using Bismark (v0.14.4) with parameters ‘-n 2 -l 50 --un --ambiguous --bowtie1 --chunkmbs 2048’, to allow for up to two mismatches and to save unaligned and ambiguously mapping reads separately. Seqtk with parameters ‘-e 3’ was used to remove the last three bases (a potential tRNA ‘CCA’ tail) from the unaligned and ambiguous reads followed by a second alignment attempt using Bismark. Finally, ngsutils (v0.5.9) in the ‘junction’ mode was used to extract splice junctions from known genes (Gencode v28) and unaligned reads from the second alignment were aligned to the junctions using Bismark. Reads aligned to the junctions were converted back to genomic coordinates using bamutil (https://github.com/statgen/bamUtil) in ‘convertregion’ mode. ‘N’ in the cigar string was replaced with ‘D’ for compatibility with bismark_methylation_extractor.

Samtools merge was used to combine aligned reads from all three alignment attempts. Reads with >1/3 methylated cytosines were discarded as they are likely artifacts from conversion-resistant regions. The bismark_methylation_extractor with the ‘--bedGraph --counts --CX_context’ options was used to extract methylated cytosines.

For comparisons with methylation sites in Huang *et al.* (11) we used the published methylation sites from their Supplementary Table S4. The liftover tools was used to convert hg19 coordinates to hg38 coordinates. However, to visualize the methylation levels in those samples, we re-aligned those datasets to the hg38 genome using our pipeline.

To detect tRNA methylation, we downloaded high-confidence tRNA annotations from GtRNAdb (http://gtrnadb.ucsc.edu, *hg38*). Some tRNAs have multiple gene copies for the same transcript sequence. We selected the first gene copy (identified by a ‘1’ in the fifth field of its name, e.g., ‘tRNA-Ala-AGC-9-1’), reducing the number of tRNA copies from 432 to 261. Next, introns were removed, the ‘CCA’ tail added, and two flanking ‘N’ on each site of the transcript was added to avoid Bismark warnings due to missing sequence context. In order not to exclude multi-mapping reads during the alignment, a genome index was prepared for each individual tRNA transcript, followed by alignment of all reads to all tRNA indices using Bismark with the parameters described above.

All valid tRNA alignments for each read were extracted. If a read mapped to multiple transcripts, one alignment among alignments with the fewest mismatches was selected at random and the others alignments were discarded. This ensures that no read is counted multiple times. Reads with >1/3 methylated cytosines were discarded as they are likely artifacts from conversion-resistant regions. Finally, the bismark_methylation_extractor with the ‘--bedGraph --counts --CX_context’ options was used to extract cytosine methylation levels.

### Quantification of methylation at miCLIP sites and known methylation sites

Methylation signal was quantified across protein-coding miCLIP sites and across the 13 published lists of methylation sites from Huang *et al.* (11) representing HEK293, HeLa (control and NSUN2 knockout) and seven tissues. Genomic coordinates were converted to hg38 using the liftover tool, and the genomic context (±10 nucleotides) of the site was extracted. For each set of sites, we analysed sites with the CTC[CT]A motif and sites without the motif separately. To reduce the noise from the miCLIP experiment, miCLIP sites located next to a motif were shifted to the motif and duplicate sites were removed. For each cytosine within 10 nucleotides from the methylation site, the total number of reads that either supported or did not support methylation were calculated. To avoid having very high-covered sites dominating the analysis, sites with more than 5 reads were normalized to 5 reads before the reads were summarized. Methylation level was calculated as the number of normalized reads supporting methylation, divided by the total number of normalized reads.

### Analysis related to translation readthrough

To determine if miCLIP sites in 3′ UTRs of protein coding genes (*n* = 522) could be associated to translation readthrough, we determined the frequency of in-frame stop codons between the nearest up-stream annotated stop codon and the 3′ UTR miCLIP site. As a control, we used a region of equal size immediately down-stream of the miCLIP site.

Translational readthrough is associated with usage or rare stop codons and unfavoured stop codon contexts, defined as the base immediately down-stream of the stop codon. The stop codon context for all annotated stop codons (Gencode V23) was retrieved using samtools faidx. Genes with multiple annotated stop codons were represented as a fraction of stop codons with sum 1. The overall stop codon context was calculated for all protein-coding genes or those that were identified as miCLIP targets, respectively.

## RESULTS

### miCLIP reveals enzymatic targets of NSUN6

RNA:m^5^C methyltransferases contain a catalytic domain with a common structural core and the S-Adenosyl methionine-binding site (Supplementary Figure S1A). Two conserved cysteines, both located within the methyltransferases active site, are required for completion of the catalytic process (31). Methylation is initiated when cysteine (C_1_) forms a covalent bond with the cytosine pyrimidine ring (32). The second conserved cysteine (C_2_) is required to then break the covalent adduct and to release the methylated RNA (Supplementary Figure S1B). Mutating C_2_ to alanine results in the irreversible formation of an enzyme-RNA crosslinked complex precisely at the methylated cytosine (Supplementary Figure S1C) (31,33,34). The cross-linked site can then be directly identified without further fixation steps during the immunoprecipitation protocol, omitting unspecific RNA-protein events (12,22). Because the crosslinking occurs during the methylation reaction, we termed the method methylation iCLIP (miCLIP) (12).

**Figure 1.**
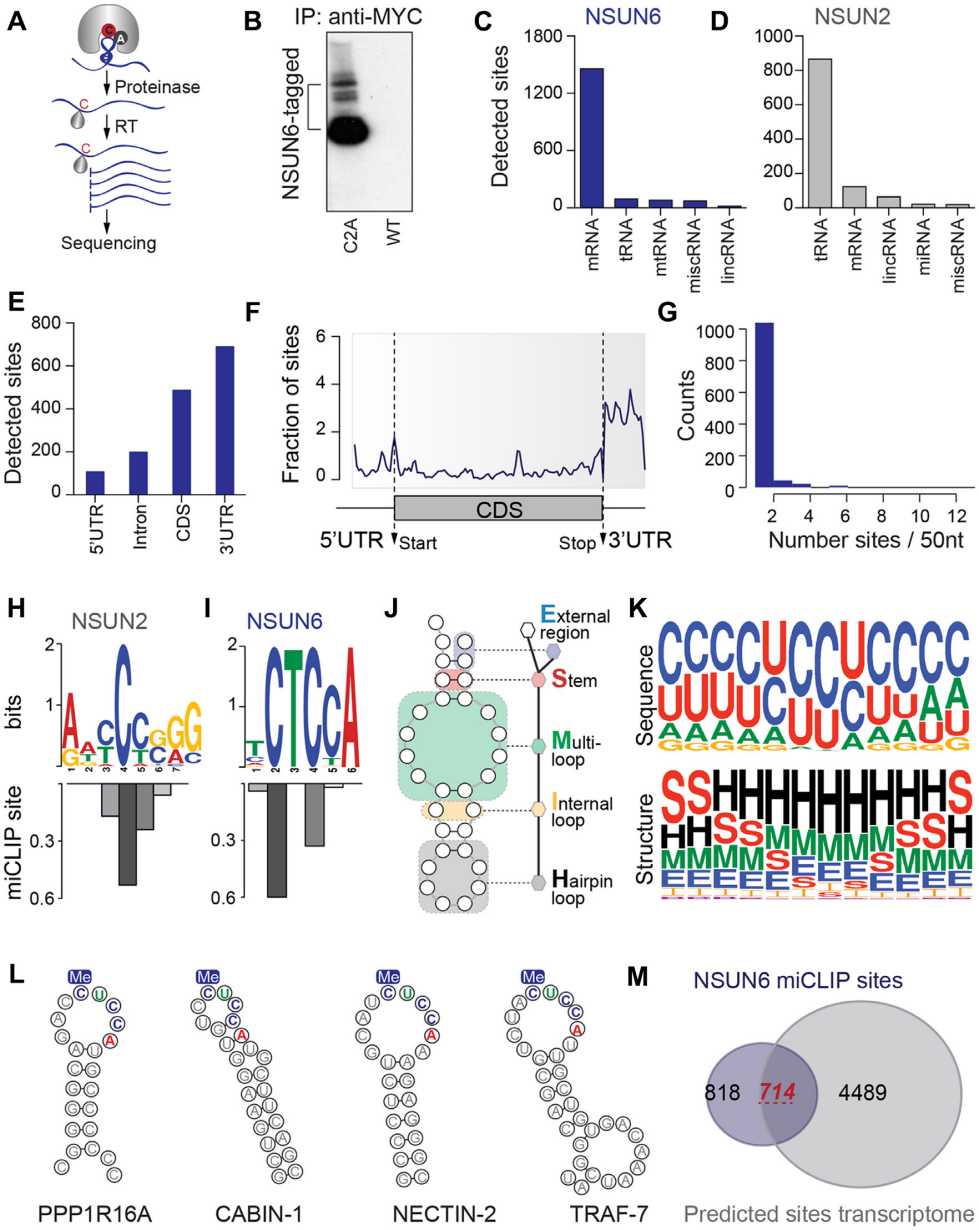
NSUN6 miCLIP reveals consensus motif in targeted RNA. (**A**) Schematic overview of the miCLIP method. (**B**) Polyacrylamide gel showing NSUN6-tagged proteins and released RNA after RNase treatment, which was isolated and sequenced. C2A: construct carrying the point mutations C→A; WT: wild-type construct. (**C, D**) Number of NSUN6 (C) and NSUN2 (D) miCLIP sites in the indicated RNAs. (**E**) Number of NSUN6 miCLIP sites in protein coding RNAs. (**F**) Distribution of NSUN6 miCLIP sites along mRNA. (**G**) Number of detected NSUN6 miCLIP sites within 50 nucleotides windows in a total of 1279 RNAs. (**H, I**) Binding motifs (upper panels) of NSUN2 (H) and NSUN6 (I) and frequency of miCLIP sites at the respective position (lower panels). (**J**) Illustration of structural motifs analysed by GraphProt. (**K**) Identified sequence (upper panel) and structural motif (lower panel) of NSUN6-targeted sites. (**L**) Examples of hairpin loop structures in NSUN6-targeted mRNAs. Me: methylation. (**M**) Overlap of predicted (grey) and observed (blue) miCLIP sites containing the sequence structure motif in the HEK transcriptome.

We previously utilized miCLIP to identify NSUN2 and NSUN3 methylated nucleosides transcriptome-wide (Figure 1A; Supplementary Figure S1A-C) (12,17). Here, we analysed a third related m^5^C RNA methyltransferase, NSUN6. We sequenced all RNAs crosslinked to the mutated NSUN6 protein (Figure 1B) and found that the majority of miCLIP sites located to mRNAs (Figure 1C; Supplementary Table S1). In contrast to NSUN6, NSUN2-specific miCLIP sites mainly occurred in tRNAs (Figure 1D; Supplementary Table S2) (12). NSUN2 and NSUN6 shared less than 4% of identified miCLIP targets (Supplementary Figure S1D), and the vast majority of shared sites in mRNAs located to non-coding RNAs or untranslated regions (Supplementary Figure S1E). Thus, NSUN6 has a distinct substrate specificity, different from NSUN2.

### NSUN6 targets 3′ UTRs in a sequence- and structure-specific manner

We previously identified that a small number of NSUN2-specific miCLIP sites in mRNAs which mostly occurred in introns of nuclear-encoded genes (Supplementary Figure S1F) (12). In contrast, NSUN6-mediated methylation occurred mainly in the 3′UTR (Figure 1E, F). The advantage of miCLIP over other RNA immunoprecipitation methods is the covalent cross-link of the enzyme to the cytosine undergoing methylation, allowing the detection of the enzymatic reaction at nucleotide-resolution (12,22). Accordingly, we found that NSUN6-specific target sites predominantly occurred as single sites (Figure 1G).

Only NSUN6-targeted sites occurred in a sequence-specific manner, and the consensus sequence motif was present in the vast majority (80%) of all targeted sites (Figure 1H, I; Supplementary Figure S1G). Since RNA secondary structures can modulate protein binding (35), we asked whether local RNA structure influenced NSUN6 targeting. We determined the sequence- and structure-specific preferences of NSUN6 miCLIP sites using GraphProt (Figure 1J) (36), and found that the methylated sites occurred preferentially centred within hairpin loops (H) of stem loop structures (Figure 1K, L). The fraction of predicted structural motifs was independent of the miCLIP threshold (10–50 RPM) (Supplementary Figure S1H). Our data is consistent with a previous study showing that NSUN2-independent methylation sites in mRNAs located to stem loop structures (11).

Finally, we asked what fraction of mRNAs contained a NSUN6-targeted sequence-structure element (Supplementary Figure S1I). First, we calculated that the minimum free energy of NSUN6-targeted RNA sequences to form a stem loop was –5 kcal/mol (Supplementary Figure S1J), and 47% of all miCLIP sites formed a stem loop under this condition (Supplementary Figure S1J). Then, we compared the predicted with the observed number of NSUN6-targeted sites in the transcriptome, and found that ∼14% of all predicted sites in mRNAs were also targeted by NSUN6 (Figure 1M).

In conclusion, mRNA targeting by NSUN6 is based on a defined sequence-structure element in mRNAs.

### RNA bisulfite sequencing confirms NSUN6-specific methylation and consensus motif

**Figure 2.**
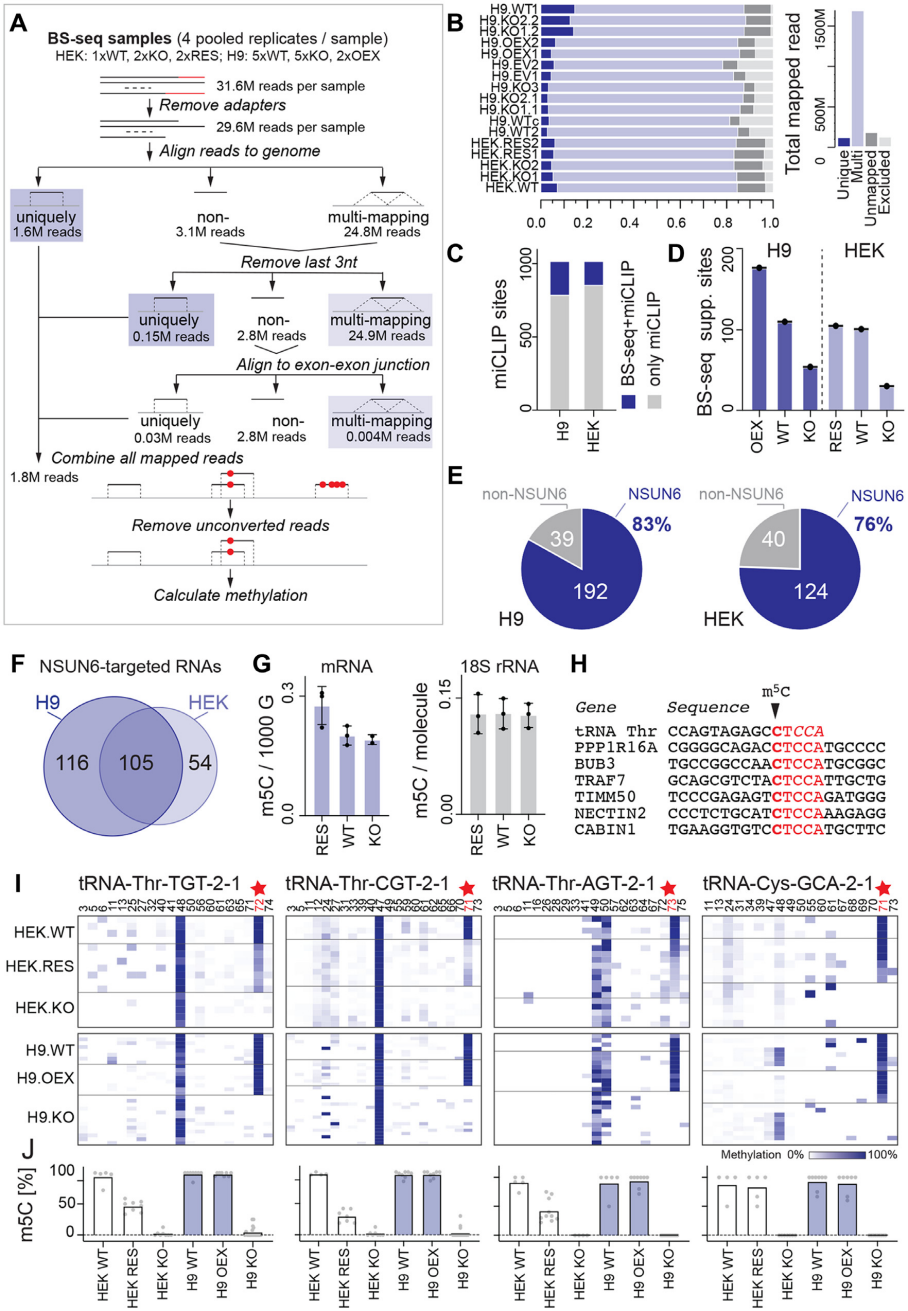
BS-sequencing confirms NSUN6 methylation sites. (**A**) Pipeline of data analyses using BS-sequencing data. (**B**) Summary of all mapped reads in the different conditions (left panel). WT: Wild-type; KO: knockout; RES: Rescue of NSUN6. Total number of mapped reads (right panel). (**C**) Overlap of miCLIP sites with m^5^C sites detected by BS-seq in H9 and HEK cells. (**D**) Methylated sites identified by miCLIP and BS-seq in the indicated conditions in HEK and H9 cells. (**E**) m^5^C sites identified by miCLIP and BS-seq (dark blue) in H9 (left panel) and HEK (right panel) cells. (**F**) Overlap of RNAs containing m^5^C sites detected by both miCLIP and by BS-seq in H9 and HEK cells. (**G**) Quantification of m^5^C in purified mRNA (left panel) and 18S rRNA (right panel) by mass spectrometry in the indicated HEK samples. (**H**) NSUN6-dependent methylation sites identified by only BS-seq in H9 and HEK cells. Red: CTCCA consensus motif. (**I**) Heatmaps of tRNA Thr and Cys isotypes carrying NSUN6-mediated methylation sites (red; star). (**J**) Quantification of (I) showing average methylation levels in the indicated samples.

The miCLIP method relies on over-expressing the mutated protein (37). To confirm endogenous NSUN6-specific methylation sites, we performed RNA bisulfite (BS) sequencing (BS-seq) (25,38). BS-seq on mRNAs remains challenging due to high RNA degradation during the protocol resulting in low mRNA coverage (39). To increase the confidence in all discovered sites, we used two independent cell lines, the human embryonic cell lines Hues9 (H9) and HEK293 (HEK). As negative controls, we generated at least two independent knockout clones via CRISPR/Cas9 genome editing for each cell line (Supplementary Figure S2A). Furthermore, we over-expressed NSUN6 in two independent H9 clones, and rescued two HEK knockout clones by stably over-expressing the NSUN6 construct (Supplementary Figure S2A). At least four replicates from each condition were subjected to the BS conversion protocol, generating a total of 68 RNA BS-seq datasets (Supplementary Figure S2A). For the analyses of the RNA BS-seq data we used our established pipeline (Figure 2A; Supplementary Table S3) (https://github.com/susbo/trans-bsseq) (21).

After mapping the reads (Figure 2B), all technical replicates were pooled to achieve the highest number of reads per cytosine in each condition. In summary, we analysed the following conditions: (i) NSUN6 knockout (KO) cells (*n* = 28), (ii) wild-type (WT) cells with endogenous expression of NSUN6 (*n* = 24), (iii) NSUN6 rescued (RES) cells (*n* = 8) and (iv) NSUN6 over-expressing (OEX) cells (*n* = 8) (Supplementary Figure S2B–E).

Next, we used the BS-seq data to evaluate the miCLIP sites, focusing on predicted sites with the sequence motif and located in protein-coding genes. As expected, the overall methylation and mRNA read coverage was often low (Supplementary Table S4). Nonetheless, the BS-seq datasets covered about 20% of miCLIP sites (Figure 2C; Supplementary Table S4). The total number of m^5^C sites detected by both methods correlated well with expression of NSUN6 (Figure 2D). The vast majority of m^5^C sites (∼80%) detected by both methods depended on NSUN6 in both cell lines (Figure 2E; Supplementary Table S4). Furthermore, the NSUN6-targeted RNAs substantially overlapped in the two cell lines (Figure 2F). Together, our BS-seq analysis reliably detected endogenous NSUN6-specific methylation sites in the transcriptome, but strongly relied on RNA abundance and methylation level at the specific cytosines.

To further confirm endogenous methylation of NSUN6-specific sites we performed mass spectrometry analysis. We revealed that only deletion of NSUN6 simultaneously with NSUN2 and DNMT2 resulted in a detectable reduction of m^5^C levels in tRNAs and large RNAs (Supplementary Figure S2F, G). Nevertheless, m^5^C levels were highest in NSUN6-rescued HEK cells in purified mRNA, but m^5^C levels in 18S rRNA was unaffected (Figure 2G). Other mRNA modifications (m^6^A and m^7^G) did not consistently change (Supplementary Figure S2H). Thus, the mass spectrometry analyses confirmed that NSUN6-dependent m^5^C sites in mRNA were present at low stoichiometry.

Next, we asked whether BS-seq confirmed the consensus motif CTCCA. All of the top seven RNAs identified by BS-seq containing NSUN6-mediated m^5^C sites, contained the CTCCA motif (Figure 2H; Supplementary Table S5). One of these sites located to tRNA^Thr^ (ACG), a previously known NSUN6-target (20). In this case, the motif was only present when taking CCA-editing of tRNAs into account (Figure 2H) (40). Since our analyses excluded multi-mapping reads (Figure 2A), we obtained poor read coverage over many tRNAs. tRNAs have low sequence uniqueness and therefore, they were not detected in our global analysis. To confirm that we can identify all known NSUN6-mediated methylation sites in tRNAs, we re-aligned our data to all tRNA genes. In this analysis, we allowed for multi-mappers. In addition to tRNA^Thr^ (ACG), we confirm tRNA^Thr^ (TGT), (CGT), (AGT) and tRNA^Cys^ (GCA) to be specifically methylated by NSUN6 (Figure 2I, J) (20). No other NSUN6-dependent m^5^C sites were found in tRNAs.

In conclusion, three independent methods identify NSUN6-dependent methylation in mRNA. However, in line with previous studies the overall number of m^5^C was low and single sites having methylation levels of >20% were extremely rare (8).

### NSUN6-specific methylation strictly depends on the consensus sequence

**Figure 3.**
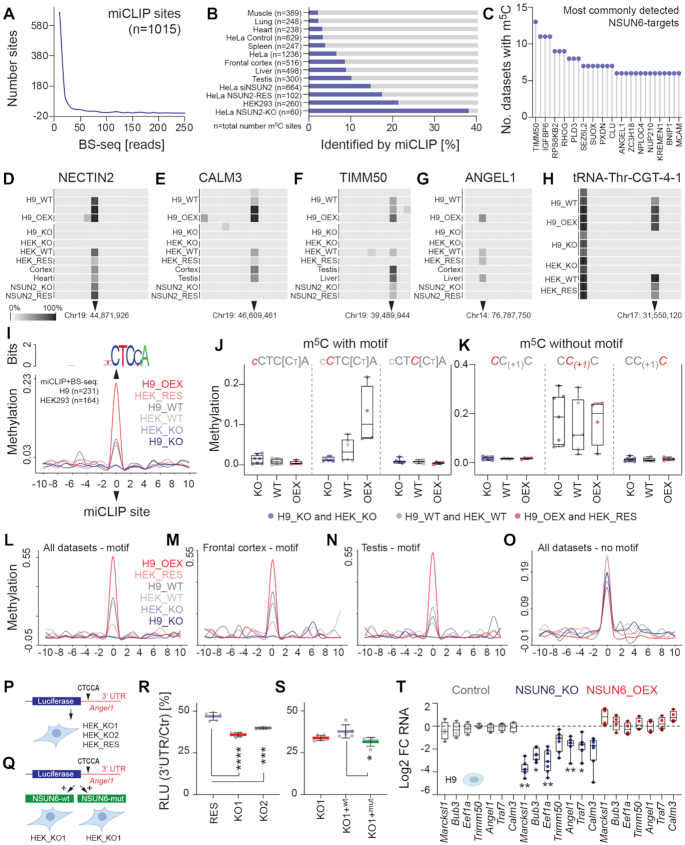
Methylation at NSUN6 consensus sequence. (**A**) Number of protein coding miCLIP sites and coverage of BS-seq reads. (**B**) Fraction of methylation sites identified in the indicated datasets overlapping with NSUN6 miCLIP sites. (**C**) NSUN6-targeted sites with confirmed m^5^C in more than 5 out of 14 different datasets. (**D–H**) Representative heatmaps showing the methylation levels in the indicated selected mRNAs (D-G), tRNA^Thr^ (CGT) as a control (H). (**I**) NSUN6-dependent methylation around the consensus sequence. (**J, K**) Mean methylation levels in knockout (KO), wild-type (WT), and NSUN6-overexpressing (OEX) cells at 994 miCLIP sites containing the consensus motif (J), and 275 sites without the consensus motif (**K**). Shown are biological replicates in H9 and HEK cells. (**L–O**) Methylation levels at published m^5^C sites (11) containing the consensus motif identified in all datasets (L) or in selected tissues of frontal cortex (M) and testis (N), or lacking the motif (O). (**P, Q**) Illustration of the luciferase reporter containing the *Angel1* 3′UTR and transfection into two NSUN6 HEK knockout clones (KO1, KO2) and one rescued KO clone re-expressing NSUN6 (P), or co-transfection with constructs expression the wild-type (wt) or methylation-deficient (mut) NSUN6 protein in HEK_KO1 (Q). (**R**) Luciferase reporter assay using constructs shown in (P). (**S**) Luciferase reporter assay using constructs shown in (Q). ****p_adj_< 0.0001; ***P_adj_< 0.001; *P_adj_< 0.05. one-way ANOVA (R,S). (**T**) RT-QPCR for selected mRNAs carrying NSUN6-methylated cytosine in control, knockout (KO) and over-expressing (OEX) H9 cells. Data are normalized to 18S rRNA and shown relative to control cells. Outlier (highest and lowest values) are removed. **P_adj_< 0.01; *P_adj_< 0.05. Two-way ANOVA (all data points).

Since NSUN6 mainly targeted mRNAs (Figure 1C), we next focused on miCLIP sites within protein coding regions. As those miCLIP sites were covered by comparably few reads in the BS-seq data (Figure 3A), we extended our analysis by asking which NSUN6 miCLIP sites were also identified in previously published datasets (Supplementary Table S4) (11,13). The miCLIP sites consistently overlapped with known m^5^C sites across 13 different cell lines and tissues, and the greatest overlap was found in NSUN2-knockout cells (Figure 3B). Thus, many of the NSUN6-dependent miCLIP sites were confirmed to be methylated in a wide range of biological conditions (Figure 3C). For instance, the m^5^C site found in translocase of inner mitochondrial membrane 50 (TIMM50) was the most commonly detected NSUN6-specific site and present in 13 out of 14 datasets, including our study (Figure 3C; Supplementary Table S4). As expected, the methylation levels of NSUN6-targeted mRNA varied between conditions, but was often supported by multiple additional datasets (Figure 3D–H; Supplementary Figure S3A). Thus, NSUN6 miCLIP reliably detected m^5^C in the transcriptome.

Next, we asked whether the consensus motif was important for NSUN6-specific methylation. NSUN6 miCLIP sites containing the motif CTC[CT]A lacked m^5^C in knockout cells, but showed enhanced methylation levels in wild-type and over-expressing cells (Figure 3I, J; Supplementary Table S6). miCLIP sites without the consensus motif displayed constant methylation levels, independent of NSUN6-expression (Figure 3K).

To further confirm that only CTC[CT]A-containing methylation sites depended on NSUN6, we analysed methylation levels in our BS-seq data across all previously identified m^5^C sites (Supplementary Table S7) (11,13). The level of methylation across published m^5^C sites also strongly correlated with expression of NSUN6, but only when the NSUN6-target motif was present (Figure 3L–O). We obtained the same results when using all identified m^5^C sites or only those detected in frontal cortex and testis (Figure 3L–O).

In conclusion, NSUN6-specific methylation of mRNA is widely present in published datasets and strictly depended on the consensus sequence motif.

### NSUN6-targeting enhances mRNA abundance and translation

To test how NSUN6 affected the targeted mRNAs, we cloned the 3′ UTR of *Angel1* into a luciferase reporter construct. First, we expressed the reporter in NSUN6 knockout (KO) and rescued (RES) HEK clones (Figure 3P). Second, we co-transfected the reporter with constructs expressing either the wild-type (wt) or methylation-deficient mutant (mut) NSUN6 protein (Figure 3Q). The reporter activity was significantly higher when NSUN6 was present (Figure 3R). Next, we asked whether the methylation activity was required to enhance the reporter activity. Re-expression of the wild-type (wt) NSUN6 protein significantly up-regulated the reporter activity when compared to the mutated (mut) NSUN6 protein (Figure 3S). Thus, NSUN6-dependent methylation enhanced the reporter activity.

To further characterize the effect of NSUN6-dependent methylation on endogenous mRNA levels, we measured the level of m^5^C containing mRNAs in the absence and presence of NSUN6 (Figure 3T; Supplementary Figure S3B). In both knockout cell lines, mRNA levels were reduced in the absence of NSUN6, but reversed to normal levels when NSUN6 expression was restored (Figure 3T; Supplementary Figure S3B), suggesting that NSUN6-mediated methylation directly or indirectly enhanced mRNA levels.

**Figure 4.**
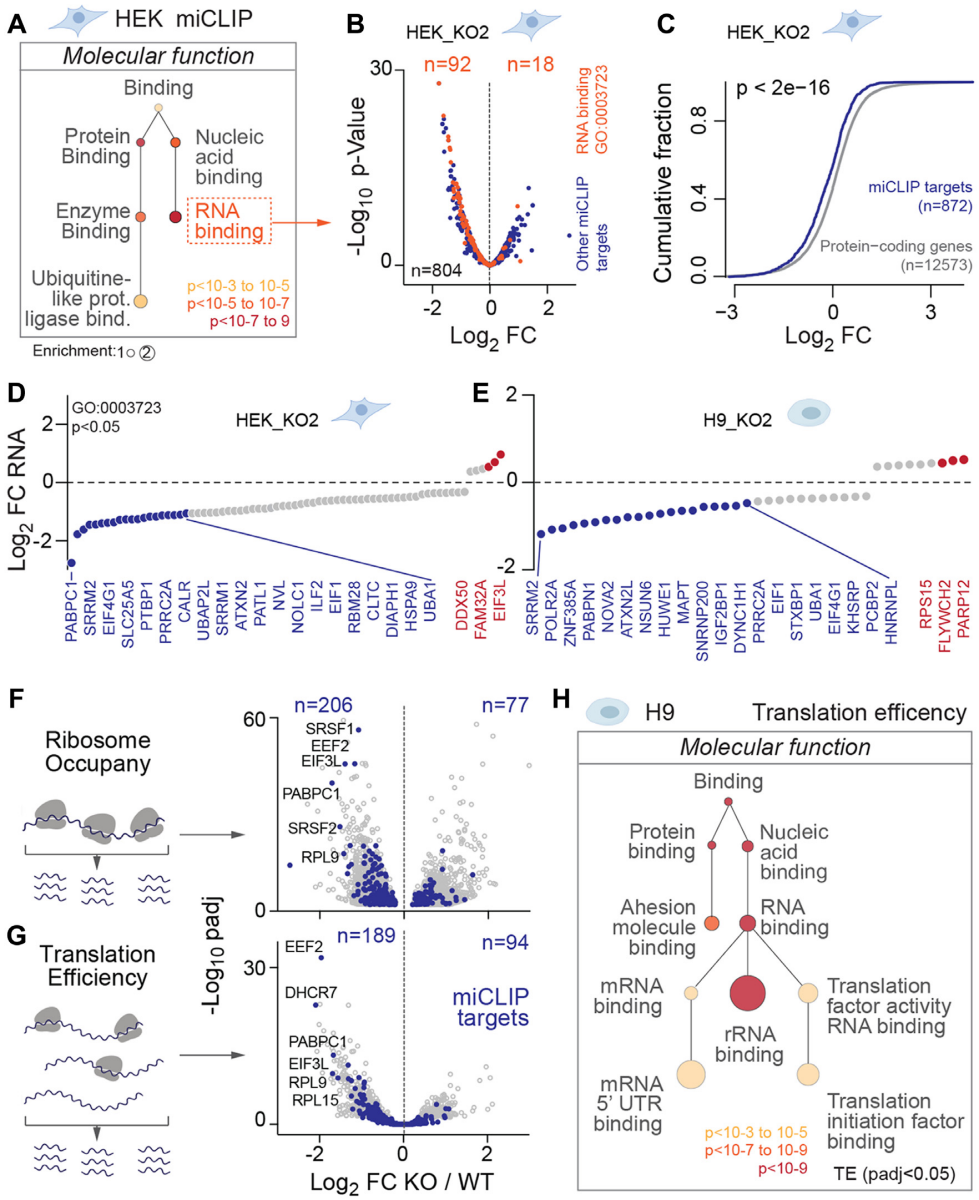
NSUN6-targeted RNAs regulate RNA processing and translation. (**A**) Gene Ontology (GO) analysis (molecular function) of all NSUN6-targeted protein coding RNAs in HEK cells using GOrilla (http://cbl-gorilla.cs.technion.ac.il/). Background: All expressed genes in HEK cells. (**B**) Log_2_ fold-change (FC) expression of all miCLIP targets. Orange dots: miCLIP targets belonging to the GO category ‘RNA-binding’ (GO: 0003723). Blue dots: All other miCLIP targets. (**C**) Cumulative fraction of log_2_ fold-change (FC) expression levels of all miCLIP targets (blue) compared to all other protein coding genes. *P*-value was calculated using the Kolmogorov-Smirnov test. (**D, E**) Examples of top up- (red) and down-regulated (blue) mRNAs targeted by NSUN6 in HEK (D) and H9 cells (E) when NSUN6 is depleted. (**F, G**) Ribosome occupancy (F) and translation efficiency (G) (illustration, left panels) of all ribosome covered mRNAs (grey) and miCLIP targets (blue) in wild-type (WT) versus NSUN6 knockout (KO) H9 cells. (**H**) Gene Ontology analysis of mRNAs with significantly (padj<0.05) changed translation efficiency.

To test whether NSUN6-targeted mRNAs shared common functions, we identified enriched gene ontology (GO) terms in all protein coding RNAs containing a miCLIP site (*n* = 906; Supplementary Table S4). NSUN6-targeted mRNAs mostly encoded for RNA- and protein-binding proteins, indicating a role in regulating gene expression (Figure 4A). Most NSUN6 miCLIP targets were down-regulated in knockout cells in RNA-seq experiments (Figure 4B, C; Supplementary Figure S4A–C; Supplementary Table S8). This was highly significant in HEK cells, where an mRNA with miCLIP sites were 5-fold more likely to be down-regulated compared with mRNAs without miCLIP sites (Supplementary Figure S4D). Repressed mRNAs encoded proteins involved in both RNA processing and translation, such as the polyadenylate-binding proteins PABPC1 and PABPN1 and the translation initiation factors EIF4G1 and EIF1 (Figure 4D, E). Thus, our data identify a function for NSUN6-targeted mRNAs in regulating post-transcriptional and translational processes (Supplementary Figure S4E). Transcriptional profiling of NSUN6-depleted HEK and H9 cells confirmed that genes encoding for RNA-binding proteins, and in particular of pre-mRNA and 3′ UTR, were significantly repressed (Supplementary Figure S4F–H; Supplementary Table S8).

Since NSUN6 mostly methylated 3′ UTRs, we speculated that deposition of m^5^C regulated mRNA translation. To test for translation differences, we performed ribosome profiling in wild-type and knockout H9 cells (Supplementary Table S9) (41). We confirmed that ribosome occupancy of NSUN6 targeted mRNAs was lower in the absence of NSUN6 (Figure 4F, blue dots; Supplementary Figure S4I; Supplementary Table S9). Since ribosome occupancy cannot distinguish between altered mRNA abundance or higher coverage of ribosomes per mRNA, we next calculated translation efficiencies. The translation efficiency of most NSUN6-targeted mRNAs was repressed (Figure 4G, blue dots; Supplementary Figure S4J; Supplementary Table S9). However, global protein synthesis was not significantly affected by depletion of NSUN6 (Supplementary Figure S4K). Again, inhibition of translation primarily affected RNA- and protein-binding factors involved in regulating mRNA processing and translation (Figure 4H). Thus, our data indicated that NSUN6 regulated gene expression on the post-transcriptional and translational level.

### NSUN6-targeted CTCCA motifs mark translation termination sites

**Figure 5.**
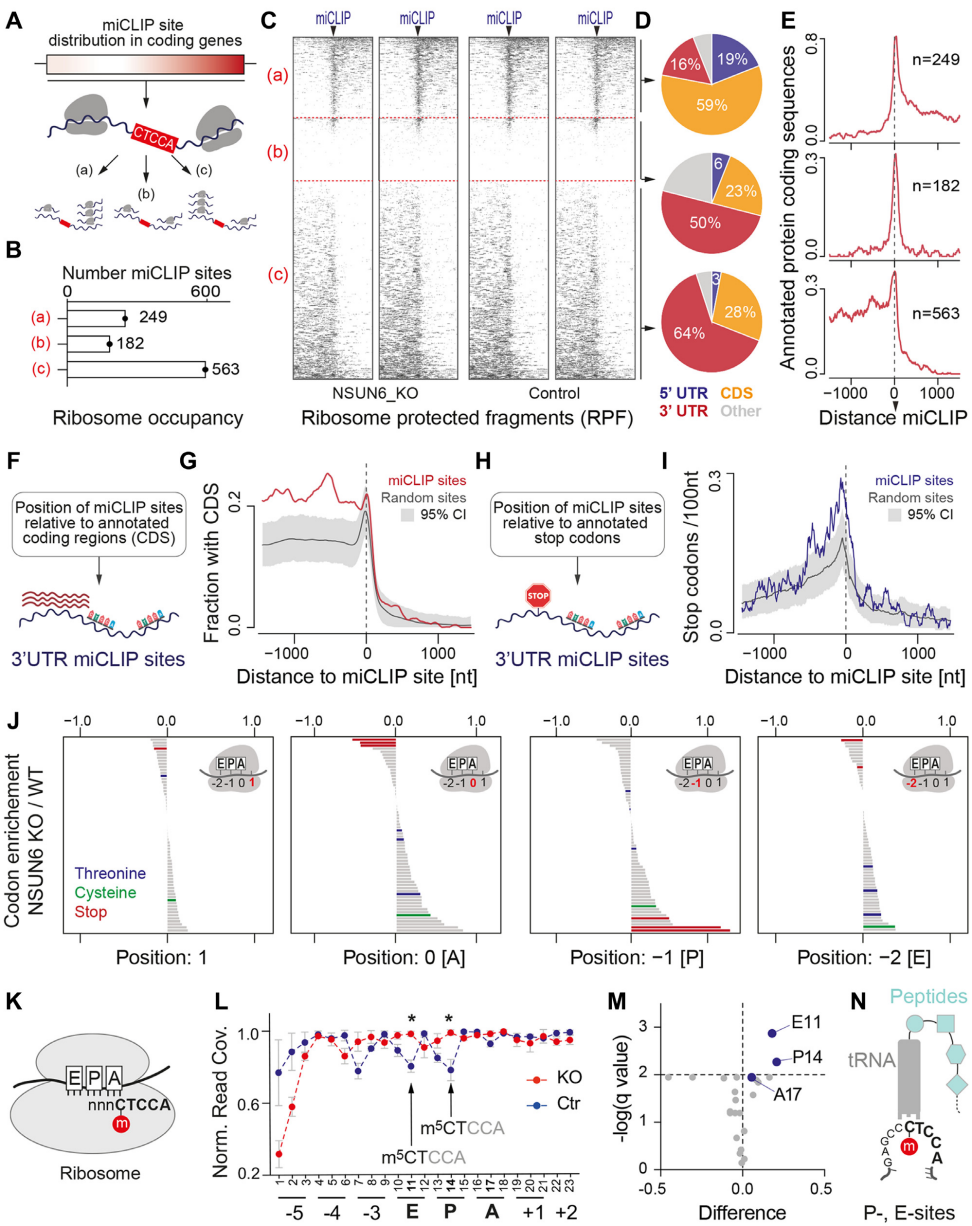
NSUN6 miCLIP sites with CTCCA consensus motif mark translation termination. (**A**) Illustration of the Ribo-seq data analysis. (**B**) Number of miCLIP sites with ribosome occupancy shown in (A). (**C**) Heatmaps of Ribo-seq reads around the miCLIP sites in NSUN6 knockout (KO) and control cells (shown are two representative replicates each). Arrowheads indicate position 0 of miCLIP sites. (**D**) Percentages of miCLIP sites located in the CDS, 5′ and 3′ UTRs in the indicated groups (a–c) (**E**) Fraction of annotated protein coding sequences with (a) lower (upper panel), (b) unchanged (middle panel), or (c) higher (lower panel) number of reads before the miCLIP sites. (**F, G**) Illustration (E) and quantification (G) of annotated CDS around 509 miCLIP or randomly selected motif sites in 3′ UTRs. The random selection was repeated 1000 times and the mean and confidence interval are shown. (**H, I**) Illustration (H) and quantification (I) of annotated stop codons around 509 miCLIP or randomly selected sites in 3′ UTRs. Random selection repeated as for (G). (**J**) Codon enrichment in NSUN6 knockout cells versus control cells at the indicated positions of the ribosomes’ active sites. Stop codons are marked red. Codons requiring NSUN6-methylated tRNAs are marked in blue and green. (**K**) Illustration of codon usage analyses covering the miCLIP consensus sequence (CTCCA). (**L–N**) Normalized read coverage with CTCCA at the indicated position of the ribosome footprint (N) with calculated significance levels (M) and illustration of codon positions at tRNAs at the P- and E-sites (N).

To determine how NSUN6-dependent methylation affected mRNA translation, we calculated the ribosome occupancy around miCLIP sites in mRNAs containing the CTCCA motif (*n* = 994) (Figure 5A). The ribosome footprints displayed a strong bias towards only one side of the CTCCA motif. Therefore, we grouped the ribosomes occupancies into (a) lower, (b) unchanged or (c) higher up-stream of the NSUN6 miCLIP site (Figure 5A,B). The majority of mRNAs had higher ribosome occupancies up-stream of the miCLIP site (Figure 5B,C), which was expected because most miCLIP sites localized to 3′ UTRs (Figure 5D). The difference in ribosome occupancy at miCLIP sites was independent of mRNA levels (Supplementary Figure S5A).

Moreover, the sharp drop in ribosome occupancy shortly after the miCLIP site in group (c), suggested translation termination. To further explore the idea that NSUN6-targeted CTCCA motifs marked translation termination, we measured the level of annotated protein coding sequences around the miCLIP site in groups (a), (b) and (c) (Figure 5E). We were surprised to see protein coding sequences directly abutting miCLIP sites in group (c), because most of those miCLIP sites overlapped with non-coding 3′UTRs (Figure 5D; Supplementary Figure S5B). To confirm this result, we repeated the analyses only considering 3′ UTR miCLIP sites (Figure 5F), revealing a significant enrichment of coding sequences up-stream of the miCLIP site, followed by a sharp drop thereafter (Figure 5G). As a control we used randomly selected 3′ UTR CTCCA sites (Figure 5G).

The absence of ribosome footprints directly after the miCLIP sites suggested stalling or removal of ribosomes. Because the miCLIP consensus sequence does not cover stop codons (Figure 1I), we asked where stop codons were positioned relative to the miCLIP sites (Figure 5H). We find annotated stop codons significantly enriched in close proximity to, but mostly up-stream of, miCLIP sites (Figure 5I). Together, our data revealed that NSUN6-targeted CTCCA sites marked translation termination.

To explore whether NSUN6 was involved in regulating translation termination, we evaluated whether depletion of NSUN6 resulted in stop codon biases during mRNA translation. When we calculated the codon frequencies at the ribosome active sites (E, P, A) we found stop codons were enriched at the ribosomal P-site in the absence of NSUN6 (Figure 5J). As the presence of a stop codon at the ribosome A-site is generally the signal to terminate protein synthesis, our data indicated altered translation termination in NSUN6-depleted cells. In contrast, we found no differences in codon usage for the NSUN6-methylated tRNAs Thr and Cys in NSUN6-depleted cells (Figure 5J).

Together, our data indicated that NSUN6-targeted CTCCA sites marked translation termination, even when these sites occurred in the 3′ UTRs (Supplementary Figure S5B). Detecting translation events in the 3′ UTR suggests that the ribosome has read through stop codons in these specific transcripts. While ribosome-profiling experiments commonly uncover stop-codon read-through (42–44), it is a very rare event (0.02% to 1.4%), and largely caused by incidental cellular errors during translation (42,43,45,46). Moreover, the rate of transcriptional read-through often depends on specific stop codons and their flanking sequences (47). While NSUN6-targeted mRNAs were not consistently enriched for specific stop codons (Supplementary Figure S5C), we measured slightly fewer alternative stops between the annotated stop and the miCLIP site when compared to nearby control sequences of the same lengths (Supplementary Figure S5D). Thus, our data suggested a potential role of NSUN6 in promoting translation fidelity by ensuring translation termination at targeted mRNAs.

To test whether NSUN6 methylation contributed to translation fidelity, we measured ribosome occupancies at CTCCA motifs (Figure 5K). In the presence of NSUN6, we found that m^5^CTCCA motifs appeared significant less often at positions 11 and 14 of the E- and P-binding sites for tRNA in the ribosome (Figure 5K–M). In these cases, the corresponding ‘CCA’ bases of the motif occupy the ribosome A- or P-site respectively (Figure 5L). Since ‘CCA’ encodes for proline, we confirmed that proline codons were significantly enriched at the ribosome A-site (Supplementary Figure S5E; Supplementary Table S10).

In conclusion, mRNAs with the codon sequence (N-m^5^C-T) followed by (C–C–A) spent less time at the ribosomes’ P- and E-site in the presence of NSUN6, possibly due to a faster removal of the ribosome (Figure 5N).

### NSUN6 is dispensable for development but might be a prognostic marker in cancer

Knockout of NSUN6 in H9 and HEK cells did not cause any apparent cellular phenotype. Since RNA modification enzymes often act directly in response to external stimuli (2), we asked whether differentiation of human embryonic stem cells was affected when NSUN6 was depleted. We differentiated the H9 cells into embryoid bodies and confirmed reduced levels of NSUN6-targeted mRNAs in embryoid bodies lacking NSUN6 (Supplementary Figure S6A). While pluripotency factors were not differentially expressed, some mesoderm markers and *Hoxa1* were consistently reduced in the absence of NSUN6 (Supplementary Figure S6B). Finally, we asked whether loss of NSUN6 affected embryonic development. To generate total knockout mice, we used two embryonic stem cell clones carrying the LacZ-reporter in exon 2 of the *Nsun6* gene leading to transcriptional disruption of a functional NSUN6 protein (Supplementary Figure S6C). Although LacZ expression revealed that *Nsun6* was quite ubiquitously expressed, we observed no gross phenotype in the absence of NSUN6 (Supplementary Figure S6D). We concluded that mouse embryonic development was largely unaffected by total loss of NSUN6.

**Figure 6.**
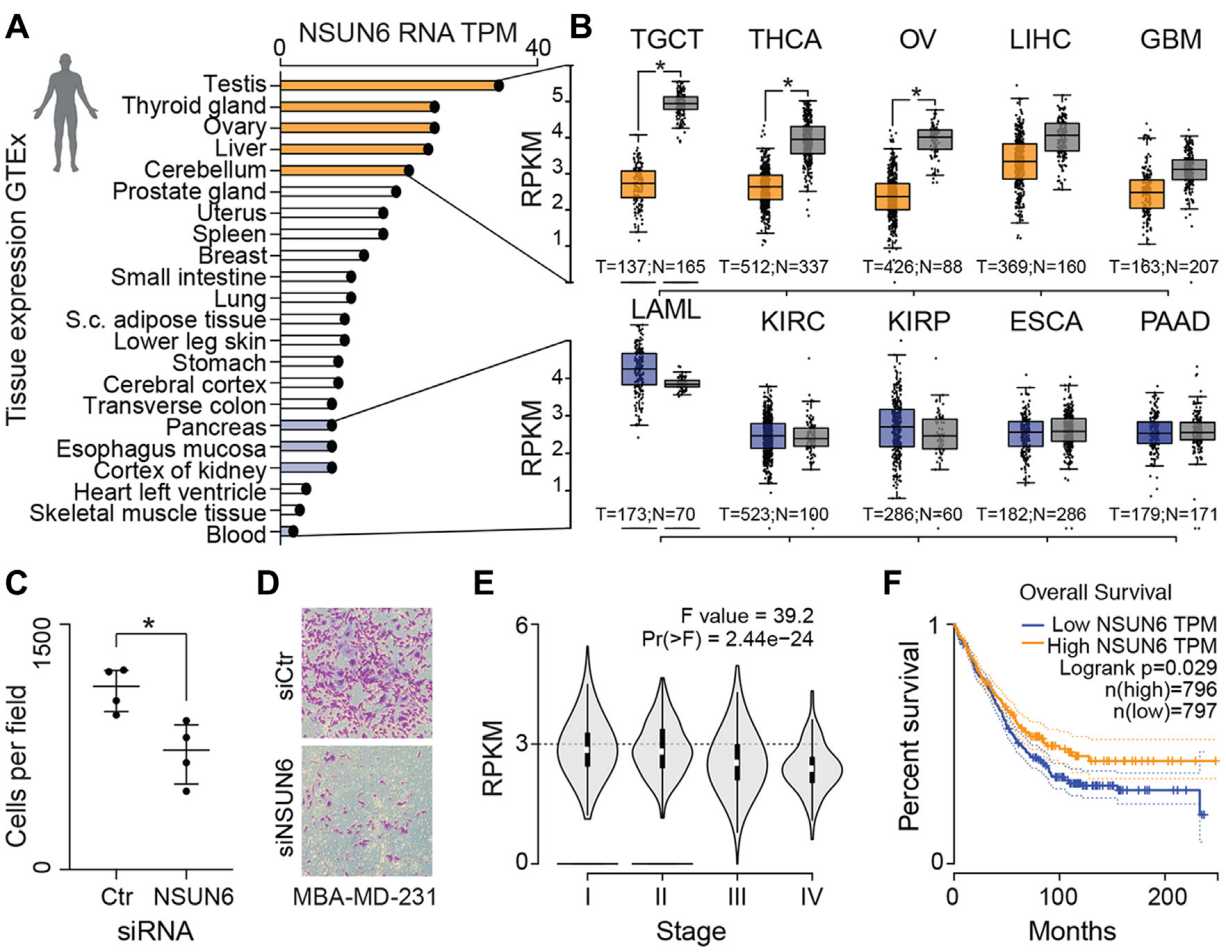
NSUN6 as a putative predictive biomarker in cancer. (**A**) *Nsun6* RNA expression levels in adult human tissues (https://www.gtexportal.org/home/). (**B**) *Nsun6* RNA expression levels in tumours derived from high (upper panel) and low (lower panel) *Nsun6* expressing tissues shown in (A) (http://gepia.cancer-pku.cn/). (**C**, **D**) Knock-down of NSUN6 (C) and migration assay (D) of cancer cells (MBA-MD-231). * *P*-value < 0.05. Unpaired *t*-test. (**E**, **F**) Stage plot (E) and survival plot (F) of *Nsun6*-low expressing cancers shown in (B; upper panel).

To identify other potential cellular functions of NSUN6, we compared *Nsun6* expression levels in human tissues collated in the Genotype-Tissue Expression (GTEx) database (Figure 6A). *Nsun6* was ubiquitously expressed with highest levels found in testis and lowest expression in blood (Figure 6A). When we analysed tumours derived from tissues with high or low *Nsun6*-expression, we found *Nsun6* mRNA levels to be down-regulated in tumours, but only when derived from high *Nsun6-*expressing tissues (Figure 6B; upper panels). *Nsun6* was significantly down-regulated in tumours derived from testis, thyroid and ovaries. In contrast, we found no difference in RNA levels when the tumours derived from low-expressing tissues, such as blood, kidney or pancreas (Figure 6B; lower panels).

In line with our finding that *Nsun6* was higher expressed in normal liver than liver tumours (Figure 6B, upper panel), over-expression of NSUN6 has recently been shown to inhibit cell proliferation of liver cancer cells (48). Cell proliferation and migration are often reciprocally controlled (49), and we find that NSUN6-expression enhanced migration in cancer cells (Figure 6C, D; Supplementary Figure S6E). However, *Nsun6*-expression was also significantly lower in later pathological stages of tumours derived from high expressing tissues (Figure 6B, upper panel; Figure 6E), and correlated with better patient survival (Figure 6F). Thus, NSUN6 might be a novel biomarker for positive patient outcome in cancers derived from testis, ovary, thyroid, liver and brain.

In summary, here we identify NSUN6 as a methyltransferase strictly targeting mRNAs at a consensus sequence motif near non-canonical translation termination sites. Loss of NSUN6-mediated methylation decreased mRNA levels and reduced translation. NSUN6 was not required for embryonic development. However, NSUN6 was down-regulated in at least some cancers and might be a novel biomarker predicting patient outcome.

## DISCUSSION

RNA modifications add flexibility, diversity and complexity to cell type and state-specific gene expression programs. Mapping these RNA modifications on single nucleotide resolution is a critical step towards understanding the underlying regulatory pathways. Despite recent advances in technologies to detect RNA modifications in mRNAs (3,50–52), the prevalence and precise location of in particular low abundance modifications remain often unclear (9). Although the need for more robust detection methods was recognised very early in the field (53), RNA BS-seq remains the method of choice for mapping m^5^C transcriptome-wide because it is currently the only available method detecting m^5^C sites on endogenous mRNAs in a quantitative manner (39).

Optimizing RNA BS-seq protocols and computational analyses is one strategy to improve the detection of m^5^C (8,11). The most recent study identified about 100 m^5^C sites per mega base in a given mammalian tissue or cell type (11). Huang *et al.* (11) further demonstrated that many but not all detected sites were NSUN2-dependent, and that NSUN2-independent sites were marked by a 3′TCCA motif. The authors suggested that this sequence motif was targeted by an uncharacterised cytosine-5 RNA methyltransferase (11). In our study, we discover the same consensus motif (CTCCA) in mRNAs and further demonstrate that it is in fact targeted by the characterised cytosine-5 methyltransferase NSUN6.

Together, our data implicate a role for NSUN6-methylation in regulating translation termination. The presence of the methyl group at CTCCA motifs in protein coding RNA correlated with greater RNA abundance and translation, while the motif itself was enriched in 3′ UTRs near sites of translation termination down-stream of annotated stop codons. Ribosome profiling further revealed an enrichment of stop codons at the ribosome P-site in NSUN6-depleted cells. Given that ribosome footprints occurred mostly up-stream, but rarely down-stream, of miCLIP sites including in 3′ UTRs, NSUN6-dependent methylation might be part of quality control mechanisms ensuring termination of translation, possibly after stop codon read-through. The low levels of stop codon read‐through may explain why the percentage of methylation is low in most NSUN6-targeted mRNAs. Our data further indicate a potential function of NSUN6 in translation termination fidelity of primarily RNA and protein-binding proteins in adult tissues of testis, ovaries and liver for example. Our finding that targeted mRNAs are more abundant in the presence of m^5^C is in line with the recent finding that m^5^C protects maternal mRNA from decay in the maternal-to-zygotic transition (54).

We find that most of m^5^C sites in the protein coding RNAs are catalysed by NSUN6. Our observation that the majority of such sites occur at low stoichiometry is one explanation why the prevalence of m^5^C in mRNAs has previously been controversial. Furthermore, the low methylation levels of mRNAs hampers studying the functional relevance of m^5^C, and may also explain the apparent lack of any gross phenotype in NSUN6-depleted mice. However, NSUN6 may confer cellular fitness advantages in specific environmental context, as we find NSUN6 to be higher expressed in healthy tissues than in tumours.

## DATA AVAILABILITY

BS-seq data are available on GEO (GSE125046). RNA-seq, Ribo-seq, miCLIP data are up-loaded onto GEO (GSE140997). The scripts used for the alignment and processing of the BS-seq data are available at [https://github.com/susbo/trans-bsseq].

## Supplementary Material

gkaa1193_Supplemental_Files
