# Supplementary material for: Sequence- and structure-specific cytosine-5 mRNA methylation by NSUN6
Source: Nucleic Acids Res. 2020 Dec 16;49(2):1006–22. doi: 10.1093/nar/gkaa1193 (PMC7826283; doi:10.1093/nar/gkaa1193)
Supplement: gkaa1193_Supplemental_Files [file gkaa1193_supplemental_files.zip › Supplementary figures 1 to 6.pdf]

Supplementary Figure 1

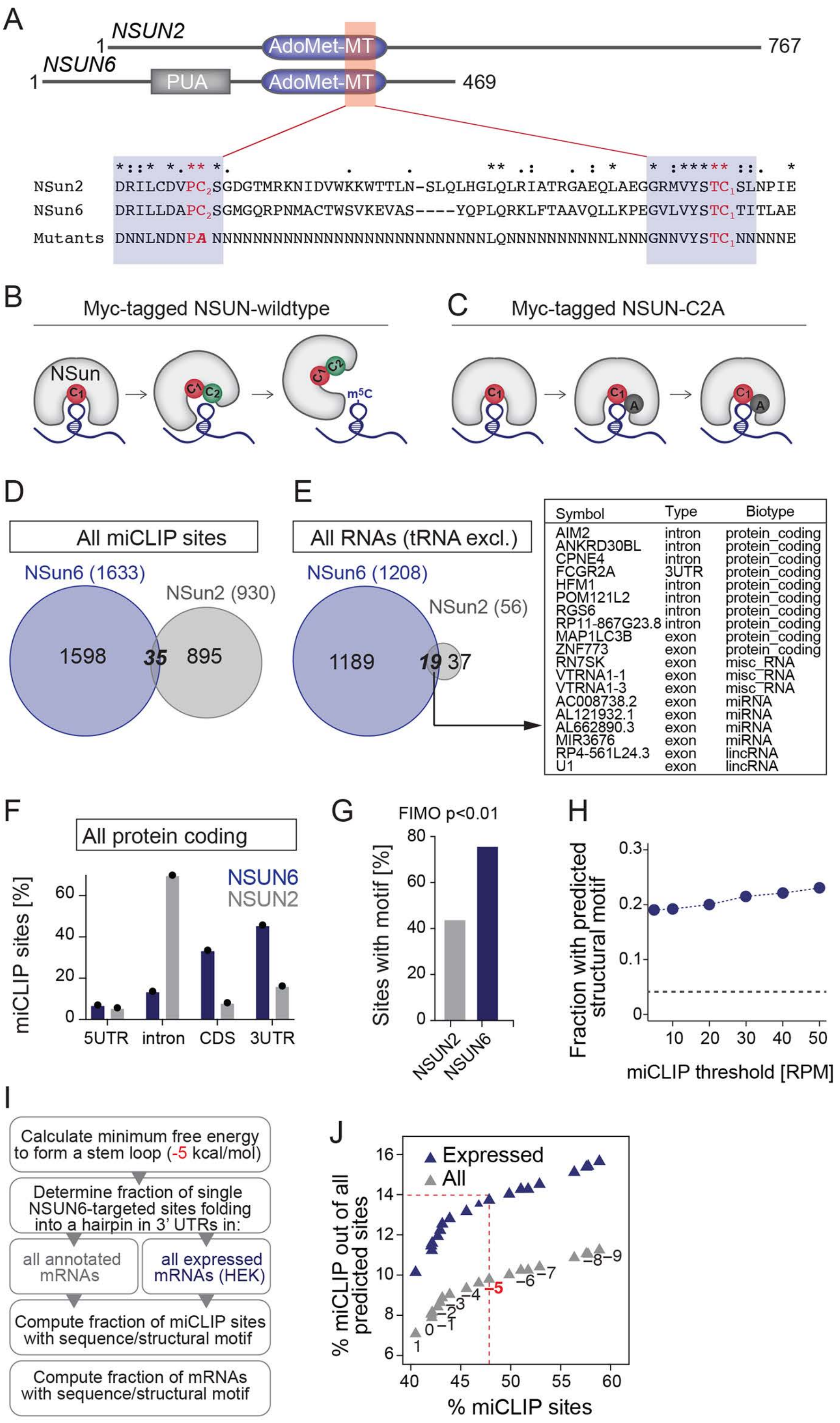

Figure S1. miCLIP identifies NSUN6-targeted RNA. (**A**) Schematic overview of conserved protein domains in NSUN2 and NSUN6 showing the Ado-Met methyltransferase (MT) superfamily domain and the amino acid sequence around the MT domain. Red letters indicate the catalytic active sites and the mutation to generate the miCLIP constructs (bold). NSUN6 contains an additional pseudouridine synthase and archaeosine transglycosylase (PUA)-domain, a conserved RNA-binding domain. (**B,C**) Overview of the mutated Myc-tagged NSUN-constructs and how they form m^{5}C (B) or the covalent bond at the methylated site (C). (**D, E**) Overlap of all NSUN2 and NSUN6 detected miCLIP sites (D) and miCLIP sites located in RNA (E). Common targets are shown in the table (right). (**F**) Percentage of NSUN2 and NSUN6 miCLIP sites in mRNAs. (**G**) Percentage of NSUN2 and NSUN6 miCLIP sites showing the consensus sequence motif shown in Figure 1H, I. (**H**) Percentage of NSUN6-targeted RNA containing sequence structure motive using different miCLIP thresholds. (**I**) Workflow to compute the number of sequence structure motifs in 3' UTRs transcriptome-wide that are targeted by NSUN6. (**J**) Comparison between miCLIP sites in 3'UTRs with free energy from 1 to -9 kcal / mol to predicted miCLIP sites in either all (grey) or expressed (blue) RNAs (> 4 log2 RPKM) in HEK cells. Red line: 47% of miCLIP sites with a free energy of -5 kcal / mol overlap with 14% of all predicted sites in expressed 3'UTRs.

Supplementary Figure 2

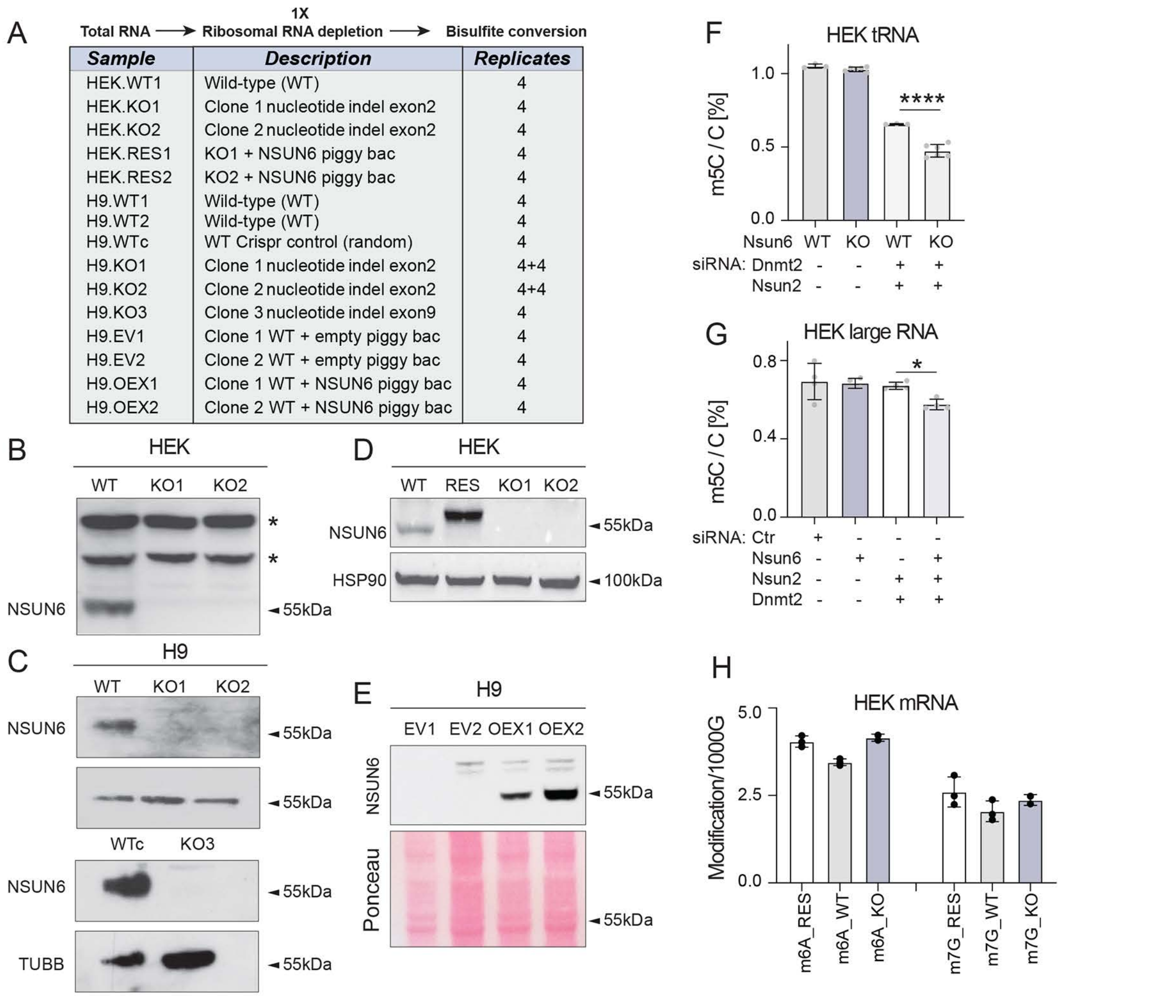

Figure S2. Sample generation used for RNA BS-sequencing. (**A**) Preparation for bisulfite conversion protocol and list of all samples analysed using RNA BS-seq. (**B**) Western blot showing loss of the NSUN6 protein in HEK cell clones. Asterisks: non-specific bands serving as internal loading controls. (**C**) Western blot showing loss of the NSUN6 protein in three H9 cell clones (KO1-3). WTc: H9 clone with random integration of an indel. Tubulin (TUBB) served as loading control (B, C). (**D**) Western blot for NSUN6 in wild-type (wt), rescued (RES), or knockout (KO) HEK cells. HSP90 served as loading control. (**E**) Western blot showing over-expression (OEX) of the NSUN6 protein in H9 cells. EV: Empty vector. Ponceau labelling of proteins served as loading control. (**F-H**) Mass spectrometry measuring m^5C in HEK cells enriched for tRNAs (F) or large RNAs (G) wild-type (WT), knockout (KO) or NSUN6 rescued (RES) HEK cells (F, H) or cells transfected with the indicated siRNAs (F, G).

Supplementary Figure 3

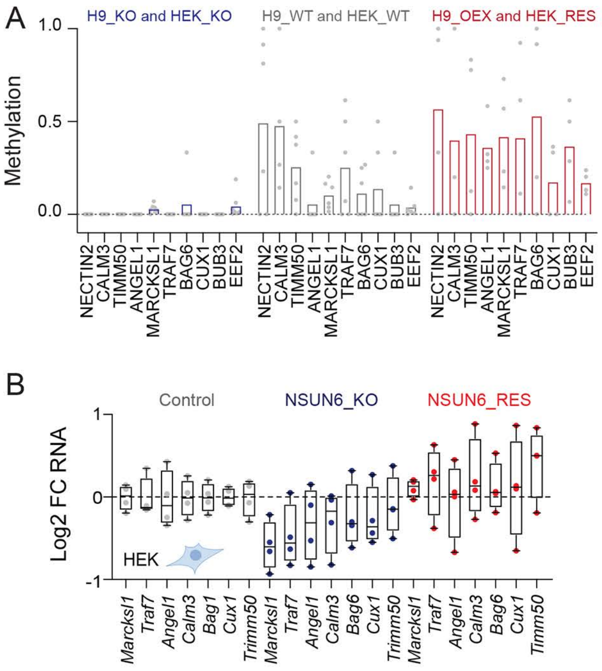

Figure S3. Methylation level of NSUN6-targeted mRNAs. (**A**) Methylation level at miCLIP sites containing the CTCCA motif in wild-type (WT) H9 and HEK cells, NSUN6 knockout (KO) cells, and NSUN6 rescued (RES) or cells over-expressing (OEX) cells. Bar plots indicate the mean methylation level in all samples. (**B**) RT-QPCR for selected mRNAs carrying NSUN6-methylated cytosine in control, knockout (KO) and rescued (RES) HEK cells. Data are normalized to 18S rRNA and shown relative to control cells.

Supplementary Figure 4

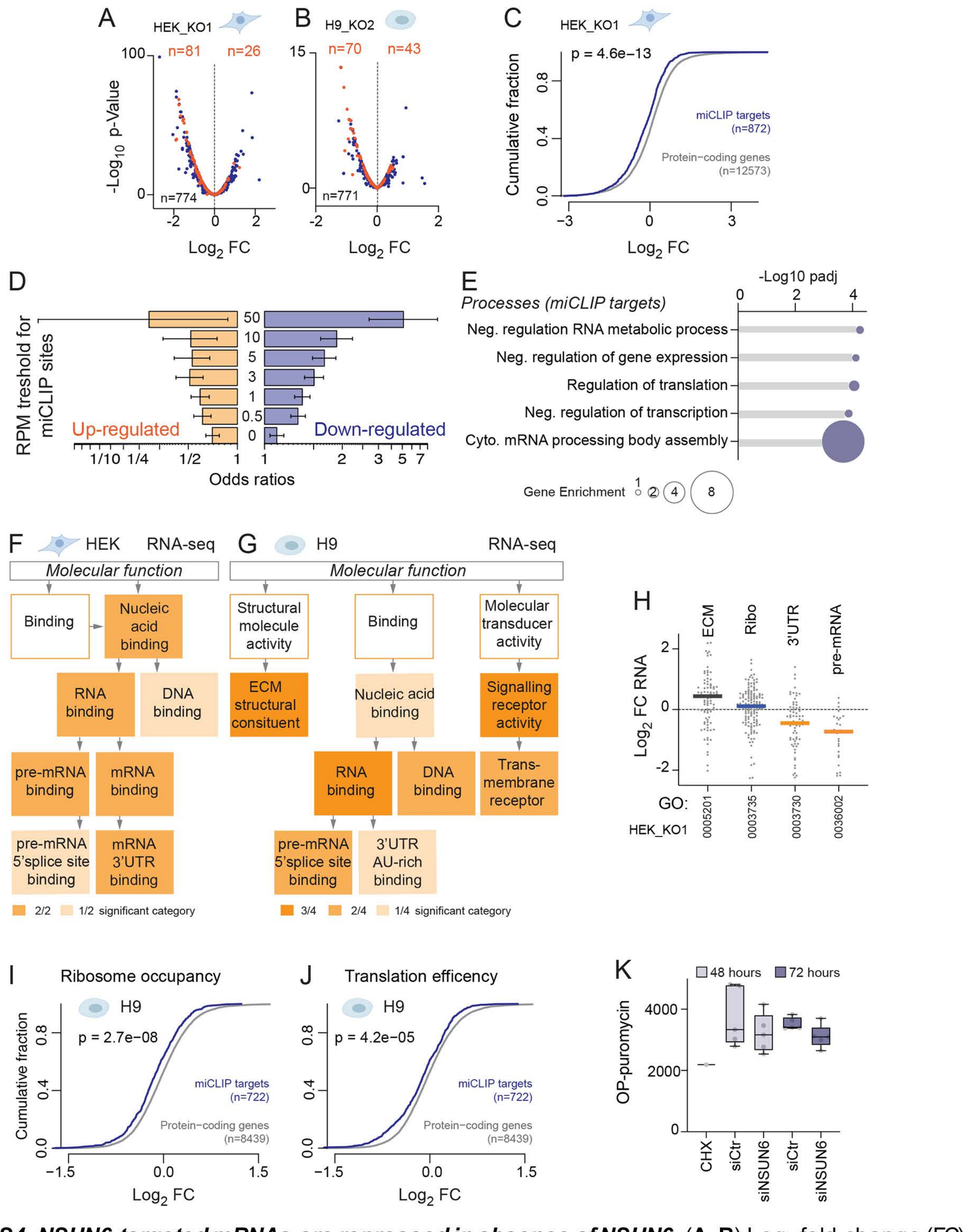

Figure S4. NSUN6-targeted mRNAs are repressed in absence of NSUN6. (**A, B**) Log_2 fold-change (FC) expression of all miCLIP targets in HEK (A) and H9 (B) NSUN6-knockout clones. Orange dots: miCLIP targets belonging to the GO category 'RNA-binding' (GO: 0003723). Blue dots: All other miCLIP targets. (**C**) Cumulative fraction of log_2 fold-change (FC) expression levels of all miCLIP targets (blue) compared to all other protein coding genes. P-value was calculated using the Kolmogorov-Smirnov test. (**D**) Odds ratios describing a higher occurrence of down-regulation (blue) and lower occurrence of up-regulation (red) in protein coding genes with miCLIP sites compared with expressed genes without miCLIP sites, at the indicated RPM thresholds. Odds ratios and 95% confidence intervals were calculated using Fisher's exact test. (**E**) Enrichment of GO terms (cell processes) of all NSUN6-targeted protein coding RNAs in HEK cells using GOrilla. (**F, G**) Gene Ontology (GO) analysis (molecular function) of NSUN6-depleted HEK (F) and H9 (G) clones using GOrilla (http://cbl-gorilla.cs.technion.ac.il/). Background: All expressed genes in HEK and H9 cells respectively. Colour code: How often the significant category was identified in the different clones (2 HEK clones; 4 H9 clones). (**H**) Transcriptional changes of genes of the indicated GO categories in HEK_KO1. ECM: Extracellular matrix; Ribo: structural constituent of ribosome. (**I, J**) Cumulative fraction of log_2 fold-change (FC) of ribosome occupancy (I) or translation efficiency (J) in knockout versus wild-type H9 cells. P-value was calculated using the Kolmogorov-Smirnov test. (**K**) Global protein synthesis rate as measured by incorporation of OP-puromycin in control (ctr) or NSUN6 siRNA transfected cells, 48 or 72 hours after transfection. Exposure to cycloheximide (CHX) served as a control.

Supplementary Figure 5

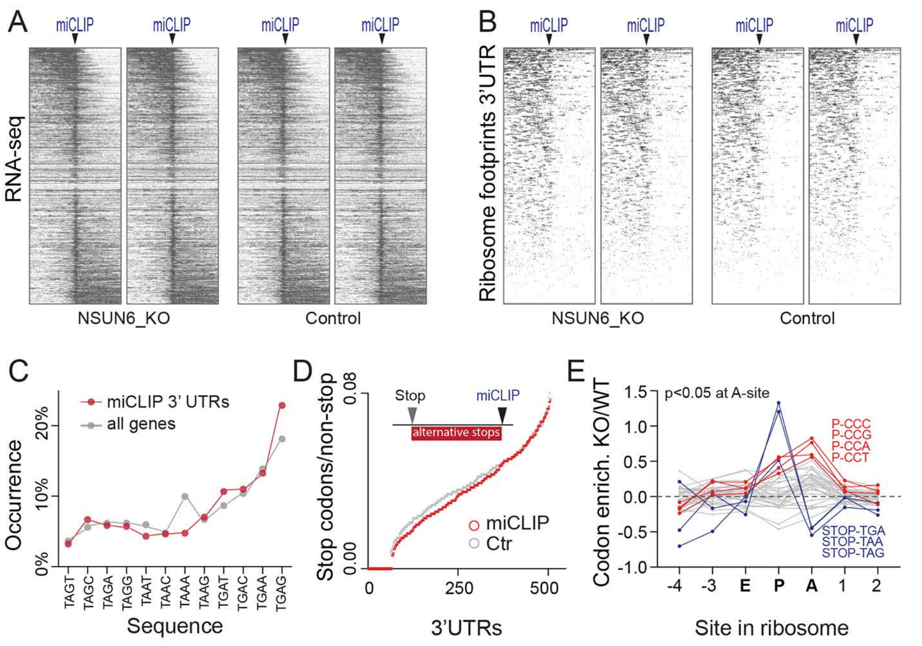

Figure S5. NSUN6-targeted CTCCA sites mark translation termination. (**A, B**) Heatmaps showing RNA-seq read coverage around the miCLIP sites (A) and ribosome footprints around 509 miCLIP sites localised in 3' UTRs (B) in NSUN6 knockout (KO) and control cells (shown are two representative replicates each). Arrowheads indicate position 0 of miCLIP sites. (**C**) Occurrence of stop codons flanked by different nucleotides in genes with 3' UTR miCLIP sites (red) compared to all genes. (**D**) Ratio between stop to non-stop codons between the annotated stop codon and miCLIP sites compared to flanking control sequences with similar lengths. (**E**) Codon enrichment at the indicated ribosome sites. Only significant different codons ($p<0.05$) are shown.

Supplementary Figure 6

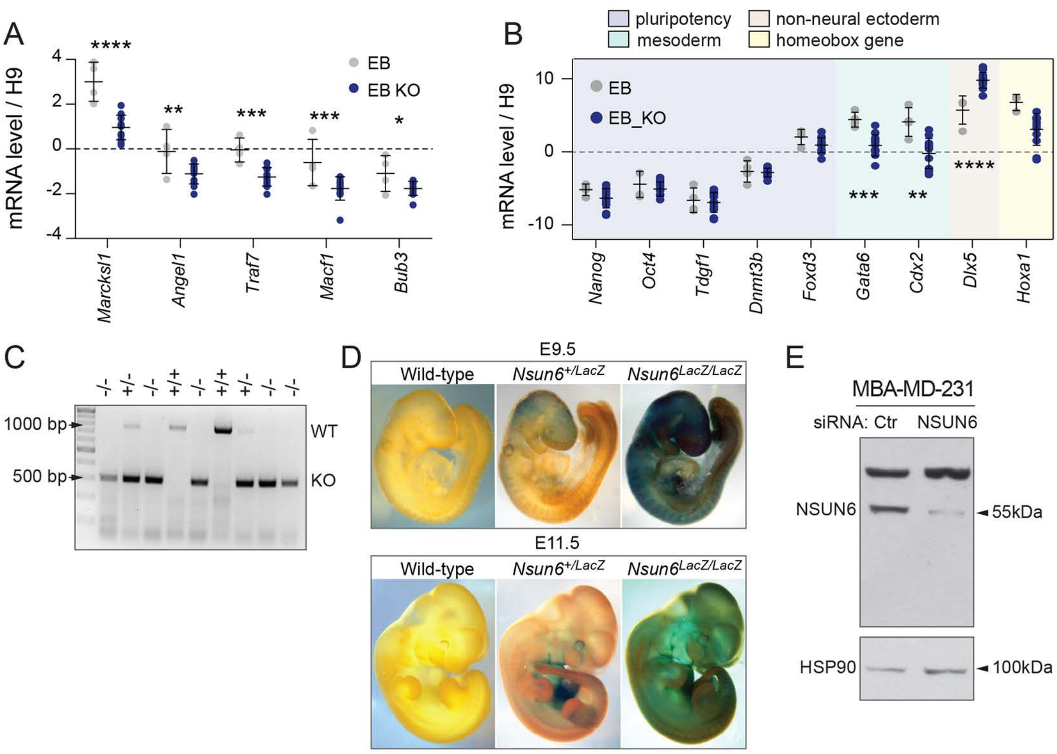

Figure S6. NSUN6 target mRNAs are down-regulated in the absence of NSUN6. (**A**) RT-QPCR for RNA for mRNAs with confirmed methylation sites in wild-type embryoid bodies (EB; 7 days) and NSUN6-depleted EB. Data are normalized to 18s rRNA and shown relative to self-renewing H9 cells. (**B**) RT-QPCR for RNA of markers for pluripotency, mesoderm, non-neural ectoderm, and a HOX gene in embryoid bodies expressing (EB) or lacking NSUN6 (EB_KO). Data are normalized to 18s rRNA and shown relative to self-renewing H9 cells. ****p<0.0001; ***p<0.001; **<0.01; *p<0.05. Multiple t-tests. (**C**) Representative PCR for genotyping *Nsun6* -/-, +/-, and +/+ mice. (**D**) Mouse embryos at the indicated embryonic (E) days are labelled for LacZ expression. (**E**) Western blot for NSUN6 in MBA-MD-231 cells transfected with a control (Ctr) or NSUN6 siRNA. HSP90 serves as a loading control.
